# Syntaxin 17 Translocation Mediated Mitophagy Switching Drives Hyperglycemia‐Induced Vascular Injury

**DOI:** 10.1002/advs.202414960

**Published:** 2025-03-26

**Authors:** Anqi Luo, Rui Wang, Jingwen Gong, Shuting Wang, Chuan Yun, Zongcun Chen, Yanan Jiang, Xiaoquan Liu, Haofu Dai, Haochen Liu, Yunsi Zheng

**Affiliations:** ^1^ School of Pharmacy China Pharmaceutical University Nanjing 211198 China; ^2^ Key Laboratory of Hainan Trauma and Disaster Rescue Hainan Medical University Haikou 571199 China; ^3^ School of Pharmacy Hainan Medical University Haikou 571199 China; ^4^ Clinical Research Center for Metabolic Disease The First Affiliated Hospital of Hainan Medical University Haikou 570102 China; ^5^ Department of Endocrinology The Second Affiliated Hospital of Hainan Medical University Haikou 570311 China; ^6^ Department of Pharmacy The Second Affiliated Hospital of Hainan Medical University Haikou 570311 China; ^7^ Hainan Key Laboratory for Research and Development of Natural Products from Li Folk Medicine Chinese Academy of Tropical Agriculture Sciences Haikou 571101 China

**Keywords:** (diabetes, Fis1, mitophagy, Syntaxin 17), vascular endothelial injury

## Abstract

The risk of diabetic cardiovascular complications is closely linked to the length of hyperglycemia exposure. Mitophagy plays a significant role in vascular endothelial injury. However, the specific mechanisms by which mitophagy contributes to endothelial injury during sustained hyperglycemia remain unclear. In diabetic ApoE^−/−^ mice and human umbilical vein endothelial cell (HUVEC) models, mitophagy is enhanced following short‐term and long‐term high‐glucose exposure. Short‐term high‐glucose exposure promotes Parkin‐mediated mitophagy and upregulates mitochondrial fission protein 1 (Fis1) expression, whereas long‐term high‐glucose exposure suppresses Parkin‐mediated mitophagy and downregulates Fis1. With prolonged high‐glucose exposure, Syntaxin 17 (STX17) translocates from the endoplasmic reticulum to the mitochondria, activating STX17‐mediated mitophagy. Silencing STX17 alleviates mitochondrial degradation, decreases reactive oxygen species (ROS) levels, enhances endothelial nitric oxide synthase (eNOS) phosphorylation, and reduces apoptosis. Silencing Fis1 accelerates the switching to STX17‐mediated mitophagy, worsening endothelial dysfunction, whereas Fis1 overexpression prevents this switching, reducing ROS and apoptosis and enhancing eNOS phosphorylation. In summary, these findings suggest that the switching from Parkin‐mediated to STX17‐mediated mitophagy drives vascular endothelial injury following long‐term hyperglycemic exposure, providing valuable insights into therapeutic strategies for diabetic cardiovascular complications.

## Graphical abstract

Mitophagy switching drives high‐glucose‐induced vascular endothelial injury. Short‐term high‐glucose exposure upregulates Fis1 expression and enhances Fis1‐TBC1D15 interaction, activating Parkin‐mediated mitophagy. Long‐term high‐glucose exposure reduces Fis1 expression, inhibiting Parkin‐mediated mitophagy and triggering mitophagy switching from Parkin‐mediated to STX17‐mediated mitophagy, leading to vascular endothelial injury. Preventing mitophagy switching may be a promising approach to alleviate high‐glucose‐induced vascular endothelial injury.

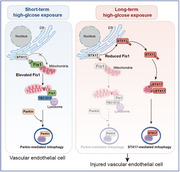

## 1 Introduction

Diabetes mellitus (DM) prevalence is rising rapidly around the world,^[^ ^1^ ^]^ with cardiovascular complications emerging as the primary cause of mortality among diabetic individuals.^[^ ^2^ ^]^ Accumulating evidence suggests that the risk of cardiovascular complications increases with prolonged glycemic exposure.^[^ ^3^ ^]^ Moreover, hyperglycemia has been shown to induce long‐lasting effects on vascular injury, as demonstrated in both experimental models^[^ ^4^ ^]^ and clinical trials.^[^ ^5^ ^]^ However, the mechanisms underlying the long‐lasting impacts of hyperglycemic exposure on complication risk have not been fully elucidated.

As the innermost layer of the vessel wall, endothelial cells (ECs) are the front line in sensing and responding to hyperglycemia.^[^ ^6^ ^]^ Endothelial dysfunction is a significant early event in the onset of diabetes vascular complications.^[^ ^7^ ^]^ Numerous studies have demonstrated that mitophagy plays a vital role in endothelial dysfunction.^[^ ^8^ ^]^ Mitophagy is a critical quality control mechanism, efficiently eliminating damaged mitochondria, maintaining intracellular homeostasis, supporting signal transduction, and mitigating ROS‐induced oxidative stress.^[^ ^9^ ^]^ Dysregulated mitophagy in ECs exposed to high glucose leads to the accumulation of dysfunctional mitochondria, triggering excessive ROS production and ultimately causing endothelial dysfunction.^[^ ^10^ ^]^ For example, high‐glucose exposure significantly inhibited mitophagy in human umbilical vein endothelial cells (HUVECs), resulting in mitochondrial dysfunction and apoptosis.^[^ ^11^ ^]^ Conversely, another study found that parkin‐mediated mitophagy increased in the vascular walls of obese mice and diabetic mice.^[^ ^12^ ^]^ Additionally, when exposed to high glucose, HUVECs treated with particulate matter significantly increased mitophagy and apoptosis.^[^ ^13^ ^]^ These discrepancies among studies may be attributed to different exposure times of high glucose. Different mitophagy mechanisms might be involved in developing endothelial dysfunction following short‐term or long‐term exposure to hyperglycemia. Thus, additional studies are required to explore and clarify the distinct mitophagy responses in ECs exposed to short‐term and long‐term hyperglycemia.

Following high‐glucose exposure, the classical Parkin‐mediated mitophagy pathway in ECs is receiving more attention.^[^ ^8^, ^14^ ^]^ A seminal finding of Parkin‐mediated mitophagy was that mitochondrial fission events at the periphery separate unhealthy daughter organelles from healthy mitochondria, with the damaged ones subsequently removed via Parkin‐mediated mitophagy.^[^ ^15^ ^]^ During this process, the enrichment of mitochondrial fission protein 1 (Fis1) regulates peripheral fission by recruiting lysosomes through its interaction with TBC1D15.^[^ ^16^ ^]^ Following peripheral fission, the membrane depolarization of daughter mitochondria recruits the E3 ubiquitin ligase parkin, leading to their clearance through Parkin‐mediated mitophagy.^[^ ^15^, ^17^ ^]^ However, this mitophagy mechanism's function in advancing high‐glucose‐induced endothelial dysfunction requires further investigation. Besides Parkin‐mediated mitophagy, Parkin‐independent pathways contribute to mitochondrial elimination, involving many proteins, including BNIP3, NIX, FUNDC1 and Fis1.^[^ ^18^ ^]^ Among these proteins, the absence of Fis1 permits STX17 to trigger mitophagy via the non‐selective autophagy machinery, independent of ectopic stimulations like mitochondrial depolarization. Under normal conditions, Fis1 functions as a gatekeeper, inhibiting the exposure of STX17's N‐terminus. The loss of Fis1 leads to the exposure of STX17's N‐terminus, thereby triggering mitophagy. Fis1‐STX17‐induced mitophagy leads to the degradation of overall mitochondrial proteins.^[^ ^19^ ^]^ Nevertheless, whether Fis1‐STX17‐mediated mitophagy is relevant to vascular endothelial injury induced by long‐term hyperglycemia exposure remains unclear.

The present study aims to elucidate the distinct impacts of short‐term and long‐term hyperglycemia exposure on vascular endothelial cells, focusing on whether distinct mitophagy mechanisms are involved. Following short‐term and long‐term high‐glucose exposure, we investigated vascular endothelial injury and its underlying mitophagy mechanisms in ApoE^−/−^ mice and HUVECs. Our findings suggest that mitophagy switching may be a critical mechanism driving high‐glucose‐induced vascular endothelial injury, and Fis1 appears to play a crucial role in mitophagy switching.

## 2 Results

### 2.1 High‐Glucose‐Induced Increase in Mitophagy Precedes Vascular Endothelial Injury

**Figure 1:**
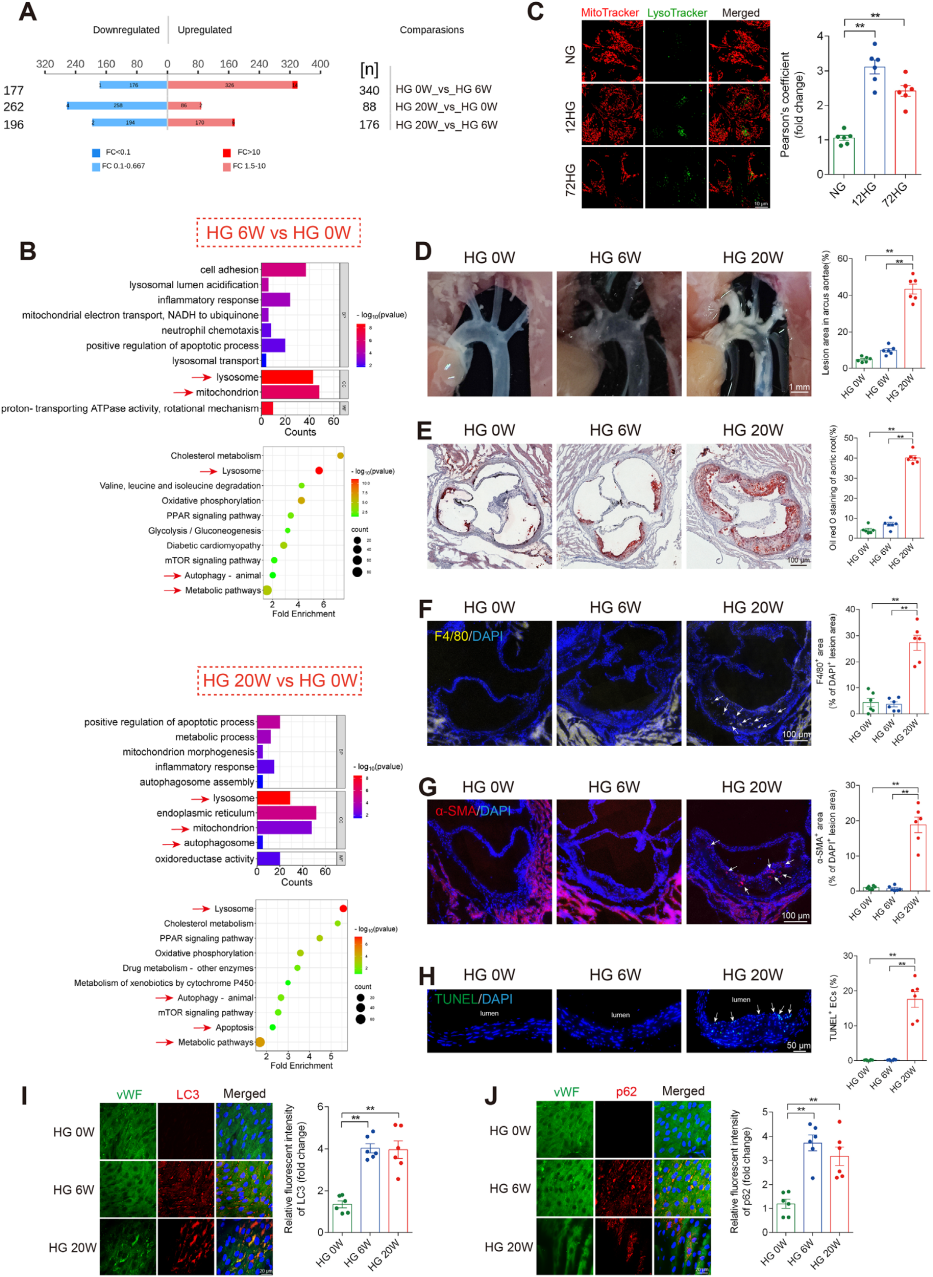
Mitophagy activation precedes hyperglycemia‐induced vascular endothelial injury. A,B, D–J) Male ApoE^−/−^ mice (6 weeks old) were injected with STZ to induce hyperglycemia. Mouse aortas were collected at 0 weeks (HG 0 W), 6 weeks (HG 6 W), and 20 weeks (HG 20 W) following hyperglycemia exposure. A,B) 4‐D label‐free quantitative proteomic analysis of aortas in mice. *n* = 3. A) Differentially expressed protein counts for the following comparisons: HG 0 W versus HG 6 W, HG 20 W versus HG 0 W, and HG 20 W versus HG 6W. B) Bar and bubble charts showing the GO and KEGG pathway enrichment analyses of differentially expressed proteins (HG 6 W vs HG 0 W, HG 20 W vs HG 0 W). C) Co‐localization of mitochondria (MitoTracker, red) and lysosomes (LysoTracker, green) in HUVECs exposed to normal glucose (NG group), 12 h of high glucose (12HG group), and 72 h of high glucose (72HG group), with Pearson's coefficient for mitochondria‐lysosome co‐localization. Scale bar = 10 µm. *n* = 6. D) Representative in situ images of atherosclerotic plaque in the aortic arch with statistical analysis. Scale bar = 1 mm. *n* = 6. E) Oil Red O staining of cross‐sections at the aortic root with statistical analysis. Scale bar = 100 µm. *n* = 6. F–G) Representative images and statistical analysis of macrophage infiltration (F4/80) F) and α‐smooth muscle actin (α‐SMA) immunostaining G) in aortic root plaque. Scale bar = 100 µm. *n* = 6. H) Representative images and statistical analysis of TUNEL^+^ cells of aortal vascular endothelium. Scale bar = 50 µm. *n* = 3. I,J) En face immunofluorescence staining of the mice abdominal aorta with immunofluorescent density analysis. *n* = 6. I) Red, green, and blue represent LC3, VWF (an endothelial cell marker) and DAPI (a nuclear marker), respectively. J) Red, green, and blue represent p62, VWF (an endothelial cell marker) and DAPI (a nuclear marker), respectively. Scale bar = 20 µm. Data are expressed as mean ± SEM. ^*^ *p* < 0.05; ^**^ *p* < 0.01.

To fully understand the mechanism of hyperglycemia‐induced vascular injury, STZ was injected into male ApoE^−/−^ mice (6 weeks old) to induce hyperglycemia. We performed a 4D‐Label free quantitative proteomic analysis by mass spectrometry of the whole aortas from three groups of mice: HG 0 W (0 weeks of hyperglycemia), HG 6 W (6 weeks of hyperglycemia), and HG 20 W (20 weeks of hyperglycemia). Through strict quality control, 4443 quantifiable proteins were obtained (Figure S1A, Supporting Information). The Venn diagrams (Figure S1B, Supporting Information) ensured the robustness and reproducibility of our data. Specifically, between HG 0 W and HG 6 W, 517 proteins showed significant differential expression, including 177 proteins that were downregulated and 340 that were upregulated. Between HG 20 and HG 0 W, 350 proteins showed significant differential expression, including 262 proteins that were downregulated and 88 that were upregulated. Between HG 20 and HG 6 W, 372 proteins showed significant differential expression, including 196 proteins that were downregulated and 176 that were upregulated (**Figure** **1**A). The heatmaps (Figure S1C, Supporting Information) illustrated distinct expression profiles of the differentially expressed proteins across the different groups. GO functional annotation and KEGG pathway enrichment analysis (Figure 1B; Figure S1D, Supporting Information) elucidated critical biological processes and pathways. In the comparison between HG 6 and HG 0 W, significant enrichment was observed in the mitochondrion, lysosome, and autophagy pathways. In the comparison between HG 20 and HG 0 W, there was marked enrichment in processes involving apoptosis, mitochondrion, lysosome, and autophagosome pathways. Comparing HG 6 and HG 20 W, alterations were evident in mitochondrion, lysosome, and metabolic pathways. Compared to HG 0 W, the differentially expressed proteins in the HG 6 W group differed from those in the HG 20 W group, but both were enriched in “mitochondrion” and ″lysosome′ pathways, which suggest that mitochondrial and autophagy‐related mechanisms may play crucial roles in hyperglycemia‐induced vascular injury. Due to limitations in observing mitochondria and lysosomes in vivo, HUVECs were stained with MitoTracker and LysoTracker in vitro, compared with the normal glucose (NG) group, 12 h (12HG) and 72 h (72HG) of high‐glucose exposure significantly increased the interaction between mitochondria and lysosomes (Figure 1C). These findings indicate mitophagy is closely linked to the mechanisms driving hyperglycemia‐induced vascular injury.

To analyze the temporal changes in mitophagy during the progression of hyperglycemia‐induced vascular injury, we measured vascular endothelial damage and the expression of autophagy marker proteins in mice at HG 0 , HG 6 , and HG 20W. The atherosclerotic lesion area was significantly increased in the HG 20 W group compared to the HG 0 W group, as shown by in situ images (Figure 1D) and Oil Red O staining (Figure 1E). This increase is accompanied by more extensive macrophage content (Figure 1F) and smooth muscle cell content (Figure 1G) in atherosclerotic lesions. Increased apoptosis in the vascular endothelium of HG 20 W group mice was detected by TUNEL staining (Figure 1H). The oxidative stress also displayed parallel changes as assessed by Immunohistochemical analysis of oxidative stress markers 3‐NT and 8‐OHdG (Figure S2, Supporting Information). In summary, the HG 6 W group showed a minor degree of vascular endothelial damage compared to the HG 0 W group, but the damage was significantly worsened in the HG 20 W group. Intriguingly, vascular en face fluorescence staining shows a significant increase in autophagy markers p62 and LC3 expression, starting at 6 weeks and persisting through 20 weeks (Figure 1I,J). These findings indicate that changes in mitophagy may occur before the onset of hyperglycemia‐induced vascular endothelial damage and oxidative stress.

**Figure 2:**
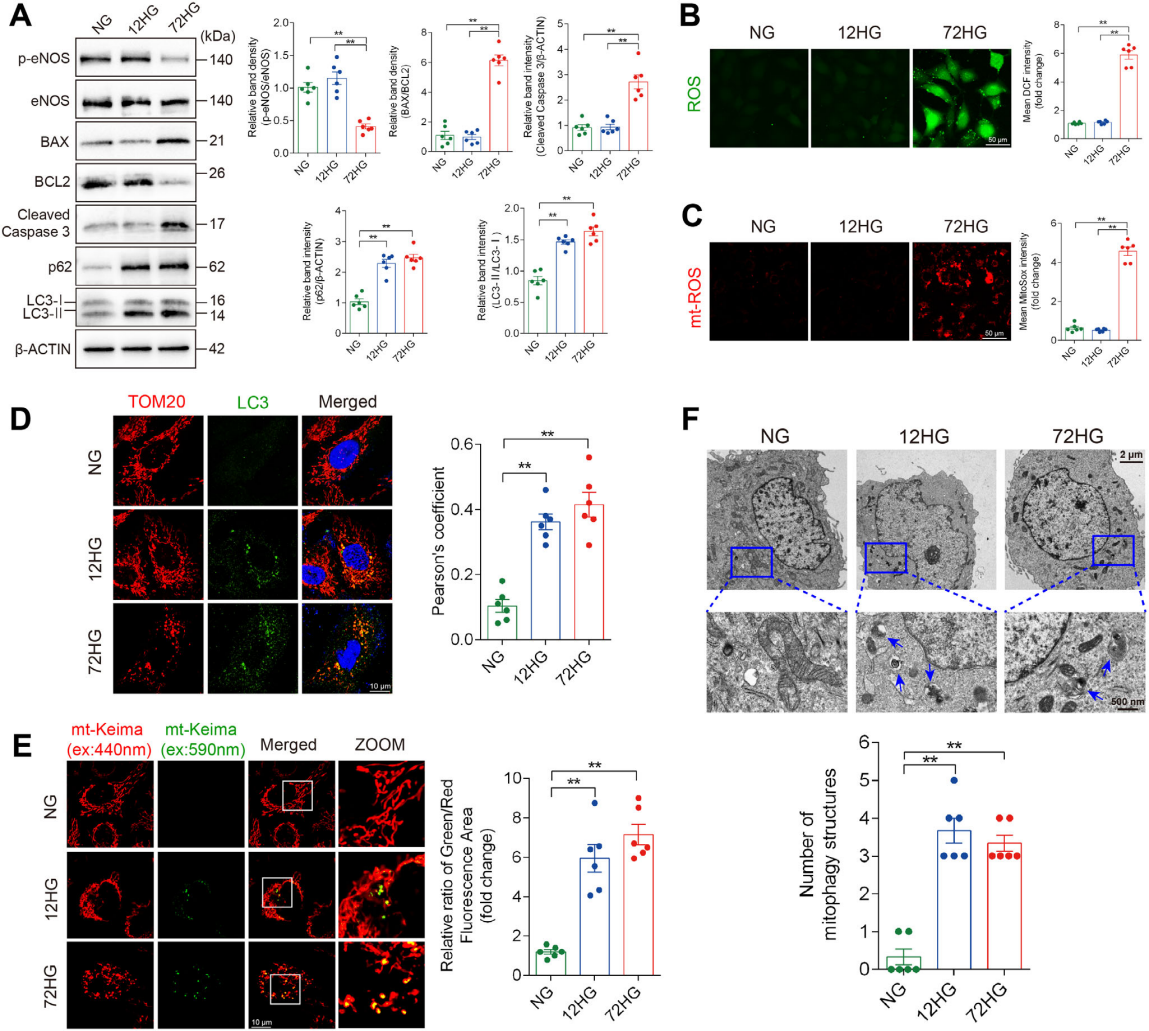
Mitophagy is activated prior to high‐glucose‐induced injury in HUVECs. A–E) HUVECs were exposed to normal glucose (NG group), 12 h of high glucose (12HG group), and 72 h of high glucose (72HG group). A) Representative Western blot images and corresponding relative intensities of BAX, BCL2, cleaved caspase 3, p‐eNOS, eNOS, LC3, and p62 bands in HUVECs. n = 6. B‐C) ROS B) and mitochondrial ROS C) production were determined by DCF and MitoSOX staining, respectively. Scale bar = 50 µm. n = 6. D) Representative IF images of TOM20 (red), LC3 (green), and DAPI (blue) in HUVECs from each group, with Pearson's coefficient for TOM20‐LC3 co‐localization. Scale bar = 10 µm. *n* = 6. E) HUVECs stably expressing mitochondrial‐targeted Keima (mt‐Keima) were imaged by confocal microscopy with excitation at 440 nm (red) and 590 nm (green). Scale bar = 10 µm. *n* = 6. F) HUVECs were analyzed by transmission electron microscopy. Blue arrows indicate autophagic structures enclosing mitochondria. Scale bar = 2 µm. *n* = 6. Data are expressed as mean ± SEM. ^*^ *p* < 0.05; ^**^ *p* < 0.01.

The level of mitophagy was also investigated during a high‐glucose‐induced endothelial injury in HUVECs, compared with normal glucose (NG group), 72 h of high‐glucose exposure (72HG group) increased the BAX/BCL2 ratio and the levels of cleaved caspase 3 expression, while reducing eNOS phosphorylation at serine 1177 (**Figure** **2**A). These changes were accompanied by increased ROS and mitochondrial ROS production (Figure 2B,C). In contrast, oxidative stress and cell apoptosis were not induced by 12 h of high‐glucose exposure (12HG group). The co‐localization of LC3 and TOM20 and ratiometric measurements of mt‐Keima (a mitochondrial‐targeted form of the fluorescent reporter Keima) were analyzed to validate mitophagy. These observations indicate that 12 h of high‐glucose exposure activated mitophagy (Figure 2D,E). Electron microscopy (EM) of HUVECs revealed a significantly increased number of mitochondria within autophagosomes in the 12HG and 72HG groups compared with the NG group (Figure 2F). This finding was further supported by increased levels of LC3 cleavage in the 12HG and 72HG groups compared with the NG group (Figure 2A). These results suggest that mitophagy is activated preceding high‐glucose‐induced vascular endothelial injury.

### 2.2 Parkin‐Mediated Mitophagy Is Activated by Short‐Term High‐Glucose Exposure but Suppressed by Long‐Term High‐Glucose Exposure

**Figure 3:**
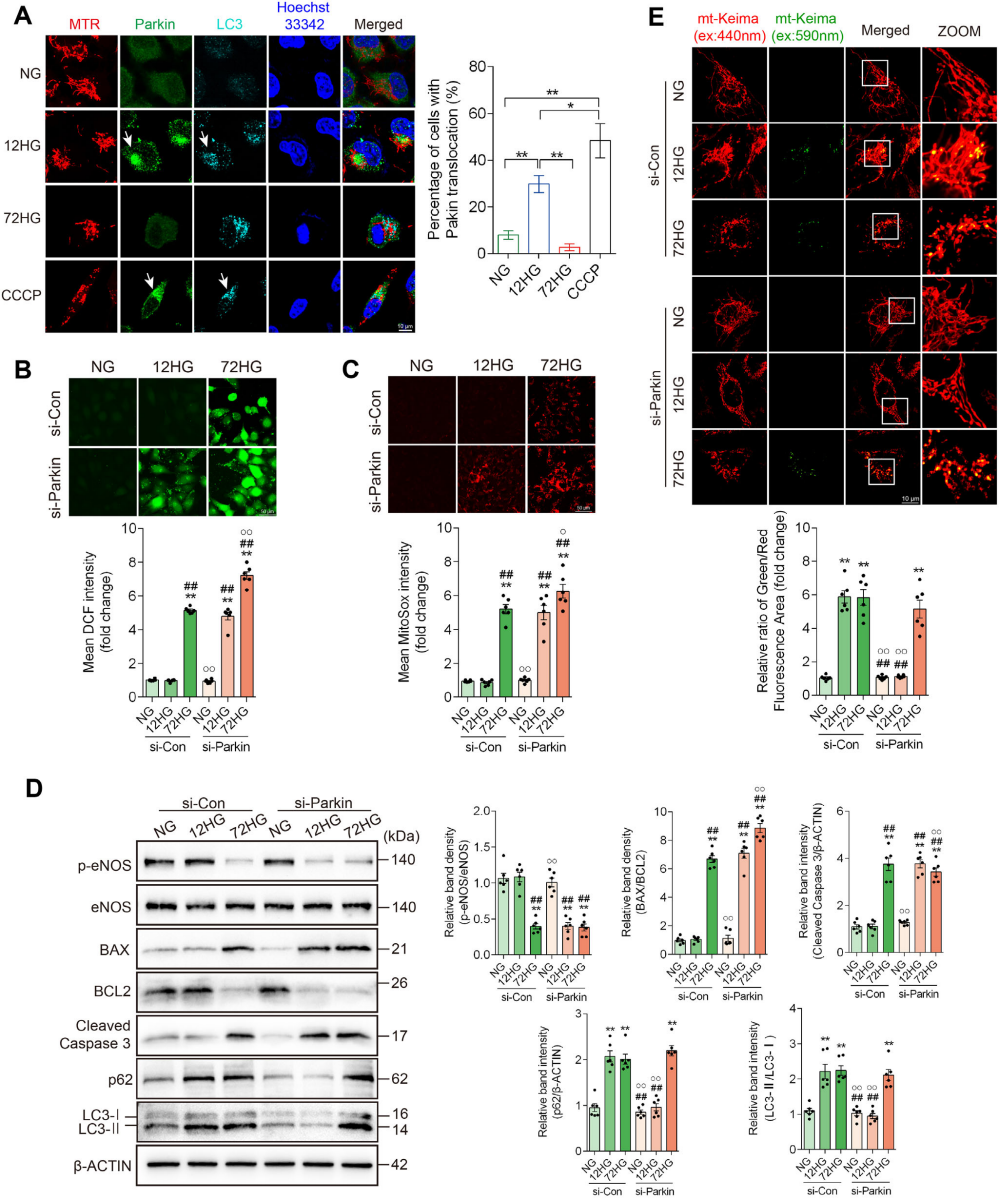
Parkin‐mediated mitophagy is activated by short‐term high‐glucose exposure while silencing Parkin accelerates and aggravates the high‐glucose‐induced vascular endothelial injury. A) HUVECs following normal glucose (NG group), 12 h of high glucose (12HG group), and 72 h of high glucose (72HG group) exposure were stained with MitoTracker (red) and analyzed by immunofluorescence microscopy for Parkin (green) and LC3 (cyan). Nuclei were stained with Hoechst (blue). HUVECs in the NG group, treated with 10 µm CCCP for 4 h, were used as a positive control. Scale bar = 10 µm. *n* = 6. B–E) HUVECs were transfected with small interfering RNA (siRNA) against control (si‐Con) or Parkin (si‐Parkin) and then exposed to normal glucose (NG group), 12 h of high glucose (12HG group), and 72 h of high glucose (72HG group). B‐C) ROS and mitochondrial ROS production were measured by DCF and MitoSOX staining, respectively. Scale bar = 50 µm. *n* = 6. D) Representative Western blot images and corresponding relative intensities of BAX, BCL2, cleaved caspase 3, p‐eNOS, eNOS, LC3, and p62 bands in HUVECs. n = 6. E) HUVECs stably expressing mitochondrial‐targeted Keima (mt‐Keima) were imaged by confocal microscopy with excitation at 440 nm (red) and 590 nm (green). Scale bar = 10 µm. n = 6. Data are expressed as mean ± SEM. A) ^*^ *p* < 0.05; ^**^ *p* < 0.01. B–E) ^*^ *p* < 0.05; ^**^ *p* < 0.01 versus NG + si‐Con group; ^#^ *p* < 0.05, ^##^ *p* < 0.01 versus 12HG + si‐Con group; ^°^ *p* < 0.05, ^°°^ *p* < 0.01 versus 72HG + si‐Con group.

Parkin‐mediated mitophagy has been considered the principal mechanism mediating mitophagy. To investigate the involvement of parkin‐mediated mitophagy in vascular endothelial injury in vitro, we monitored the co‐localization of Parkin, mitochondria, and LC3. Compared with normal glucose (NG group), 12 h of high‐glucose exposure induced the mitochondrial translocation of Parkin. However, no Parkin translocation was detected in the 72HG group (**Figure** **3**A). HUVECs treated with HG were transfected with or without Prkn‐targeting siRNA (si‐Parkin) (Figure S3A,B, Supporting Information). Oxidative stress and apoptosis were elevated, while eNOS phosphorylation was decreased in 12‐h HG‐treated si‐Parkin HUVECs compared to 12‐h HG‐treated control HUVECs (Figure 3B–D). Moreover, apoptosis and oxidative stress were further exacerbated in 72 h HG‐treated si‐Parkin HUVECs, as reflected by an increased BAX/BCL2 ratio, ROS, and mitochondrial ROS production (Figure 3B–D). These observations suggest that high‐glucose‐induced vascular endothelial injury was accelerated and aggravated by si‐Parkin, indicating that Parkin‐mediated mitophagy plays a prominent role in protecting against endothelial injury. Furthermore, long‐term high‐glucose exposure inhibited Parkin‐mediated mitophagy, potentially leading to ROS overproduction, endothelial dysfunction, and apoptosis in HUVECs.

The elevated overall mitophagy was reduced by si‐Parkin following 12 h of high‐glucose exposure in HUVECs, indicating that Parkin‐mediated mitophagy is the predominant mitophagy pathway activated by short‐term high‐glucose exposure. However, it was not suppressed by si‐Parkin in the 72HG group, as reflected by mt‐Keima (ex:590 nm) fluorescence ratio and levels of LC3 cleavage (Figure 3D,E). The results suggest that endothelial cells exposed to long‐term high glucose inhibit Parkin‐mediated mitophagy and activate additional Parkin‐independent mitophagy pathways.

### 2.3 Long‐Term High‐Glucose Exposure Initiates STX17‐Mediated Mitophagy

**Figure 4:**
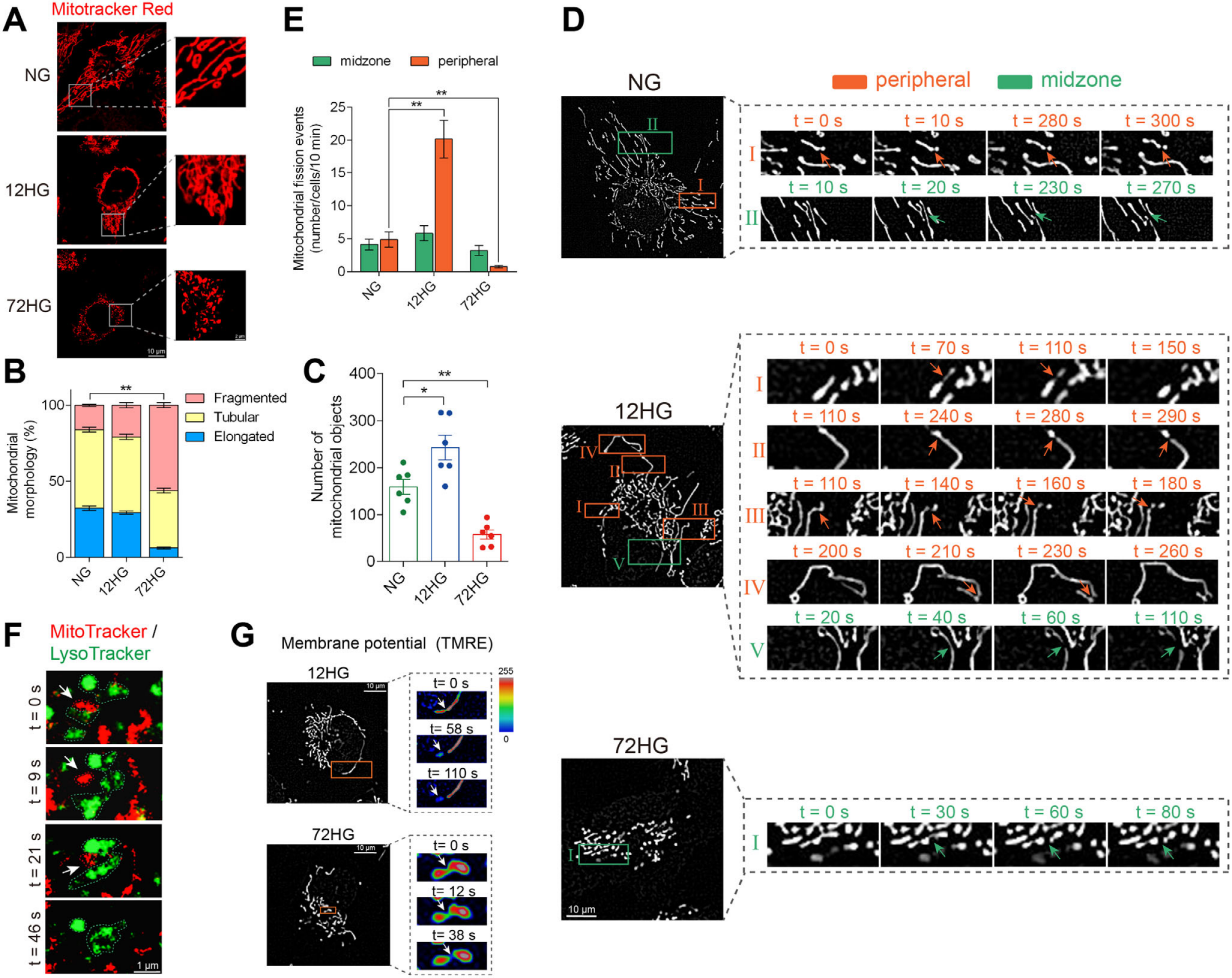
Long‐term high‐glucose exposure activates a mitophagy pathway independent of mitochondrial depolarization. A–F) HUVECs were exposed to normal glucose (NG group), 12 h of high glucose (12HG group), and 72 h of high glucose (72HG group). A) HUVECs were stained with MitoTracker Red. Mitochondrial length B) and total number of fragmented, tubular, and elongated mitochondrial structures C) were quantitatively analyzed in each group. Scale bar = 10 µm. *n* = 6. D) The mitochondrial morphology of live HUVECs stained with MitoTracker Red was recorded using time‐lapse confocal microscopy, capturing images at 10s intervals over 10 min. Scale bar = 10 µm. E) Quantification of mitochondrial fission events in live HUVECs was performed, with peripheral fission events (orange, 0–25%) occurring near the tip and midzone fission events (green, 25%–50%) near the center. n = 20. F) Positioning of mitochondria (MitoTracker, red) and lysosomes (LysoTracker, green) in HUVECs exposed to 72 h of high glucose. Scale bar = 1 µm. G) The mitochondrial membrane potential was assessed during peripheral and midzone fission in TMRE‐stained mitochondria in the 12HG and 72HG groups. Scale bar = 10 µm. Data are expressed as mean ± SEM. ^*^ *p* < 0.05; ^**^ *p* < 0.01.

Short‐term and long‐term high‐glucose exposure may activate distinct mitophagy pathways. To test this, daughter mitochondria resulting from mitochondrial fission were first tracked, and their distinct mitophagy fates were observed. During mitophagy, dysfunctional portions of mitochondria separate from healthy portions through mitochondrial peripheral fission (occurring less than 25% from a tip). Daughter mitochondria, resulting from peripheral fission and loss of mitochondrial membrane potential (membrane depolarization), are subsequently targeted for mitophagy.^[^ ^15^, ^20^ ^]^ HUVECs were imaged following 12 h of high‐glucose exposure, and elevated peripheral fission rates were observed compared to the NG group, whereas the midzone fission rates were unaffected. However, 72 h of high‐glucose exposure reduced the rates of peripheral fission, while the midzone fission rate remained constant (**Figure** **4**D,E). HUVECs exposed to long‐term high‐glucose showed inhibited peripheral fission and did not produce daughter mitochondria with membrane depolarization (Figure 4G). It seems inconsistent with the mitophagy activation observed in the 72HG group. Additionally, mitochondria in the 72HG group were entirely enveloped by LysoTracker‐labeled lysosomes, leading to their subsequent degradation (Figure 4F). This process may contribute to mitochondrial fragmentation and loss (Figure 4A–C). Therefore, we speculated that endothelial cells exposed to long‐term high‐glucose may activate an additional, non‐selective mitophagy pathway independent of membrane depolarization.

**Figure 5:**
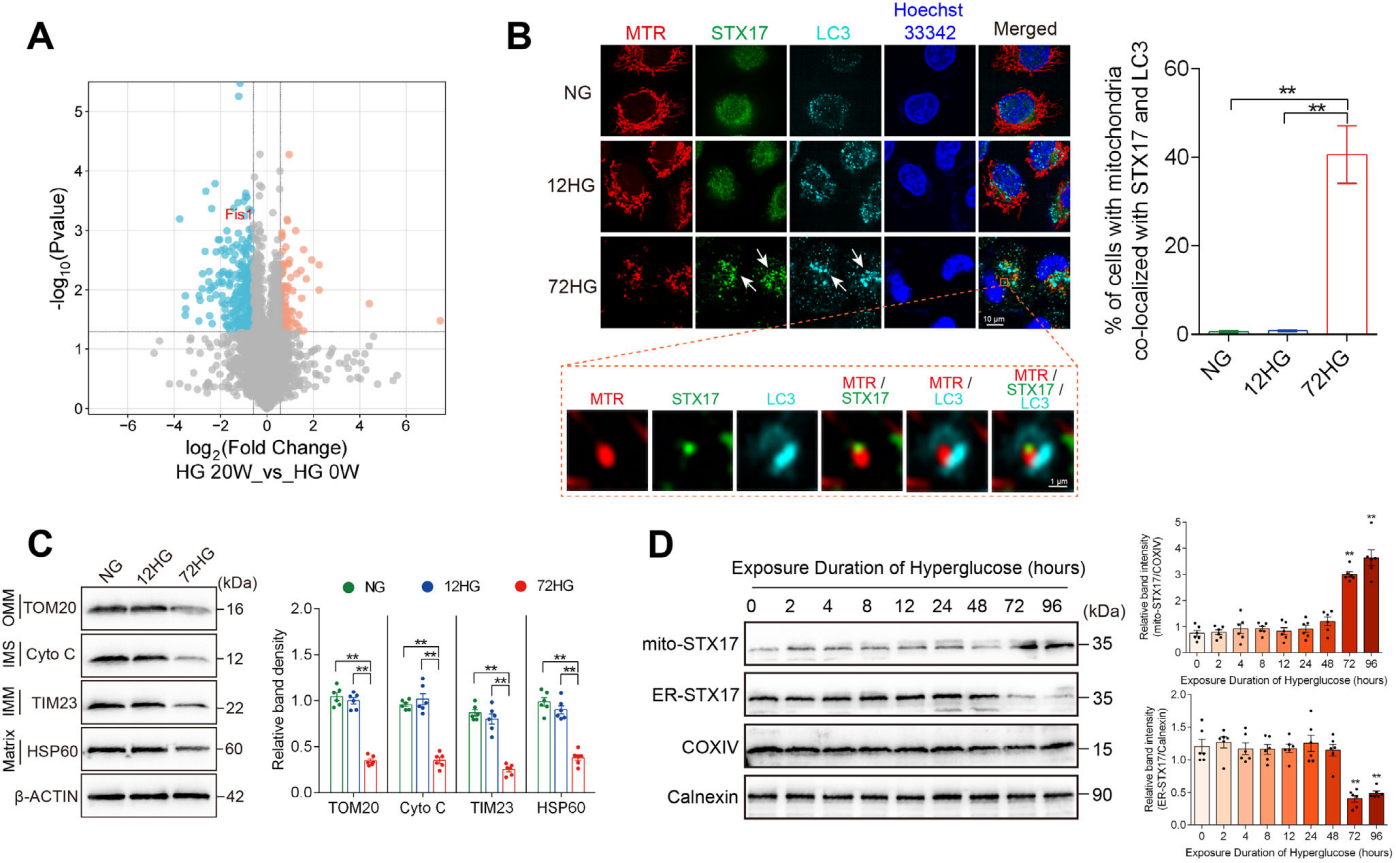
Long‐term high‐glucose exposure triggers STX17‐mediated mitophagy in HUVECs. A) Male ApoE^−/−^ mice (6 weeks old) were injected with STZ to induce hyperglycemia. Mouse aortas were collected at 0 weeks (HG 0 W) and 20 weeks (HG 20 W) following hyperglycemia exposure. Volcano plot (HG 20 W versus HG 0 W) showing differentially expressed proteins, with downregulated Fis1 labeled. B) HUVECs were exposed to normal glucose (NG group), 12 h of high glucose (12HG group), and 72 h of high glucose (72HG group). HUVECs were labeled with MitoTracker (red) and analyzed using immunofluorescence microscopy to detect STX17 (green) and LC3 (cyan), with nuclei stained with Hoechst (blue). Scale bar = 10 µm. An enlarged image of STX17‐mediated mitophagy in the 72HG group is shown. Scale bar = 1 µm. *n* = 6. C) Representative Western blot images and corresponding relative intensities of TOM20, Cyto C, TIM23, and HSP60 bands, representing proteins from the OMM, IMS, IMM, and matrix. OMM is the outer mitochondrial membrane, IMS is the intermembrane space, and IMM is the inner mitochondrial membrane. *n* = 6. D) Representative Western blot images and corresponding relative intensities showing temporal changes in STX17 expression in mitochondria and the endoplasmic reticulum (ER) following high‐glucose exposure, with COXIV used as a mitochondrial marker and Calnexin as an ER marker. n = 6. Data are expressed as mean ± SEM. ^*^ *p* < 0.05; ^**^ *p* < 0.01.

Parkin‐independent and non‐selective mitophagy pathways mediated by proteins such as Nix, FUNDC1, Bcl‐2‐L‐13, FKBP8, and Fis1 have been reported.^[^ ^18^, ^21^ ^]^ Among these, 4D‐label‐free quantitative proteomic analysis revealed that Fis1 was the only protein with significantly downregulated expression in the aorta of mice exposed to 20 weeks of hyperglycemia (**Figure** **5**A). Previous studies suggested that loss of Fis1 facilitates STX17 translocation from the endoplasmic reticulum (ER) to mitochondria, which allows STX17 to trigger mitophagy via the non‐selective mitophagy machinery.^[^ ^19^, ^21^ ^]^ To verify whether long‐term high‐glucose exposure triggers STX17‐mediated mitophagy, the percentage of mitochondria co‐localized with STX17 and LC3 in HUVECs was evaluated. Compared to normal glucose (NG group), 72 h of high‐glucose exposure significantly increased Fis1‐STX17‐induced mitophagy in HUVECs. Fragmented mitochondria co‐localized with STX17 were observed to be enclosed within LC3‐labeled autophagosomes (Figure 5B). Additionally, to monitor temporal changes in the subcellular distribution of STX17 during high‐glucose‐induced endothelial injury, mitochondria and ER were extracted and identified using organelle markers (COXIV for mitochondria and Calnexin for ER). STX17 expression was continuously measured in HUVECs, showing that following 72 h of high‐glucose exposure, STX17 levels decreased in the ER while increasing in the mitochondria, indicating its translocation from the ER to mitochondria (Figure 5D). These results suggest that long‐term high‐glucose exposure initiates STX17‐mediated mitophagy in HUVECs, causing a significant reduction in overall mitochondrial protein levels, as detected by immunoblotting (Figure 5C).

### 2.4 Silencing STX17 Alleviates High‐Glucose‐Induced Vascular Endothelial Injury

**Figure 6:**
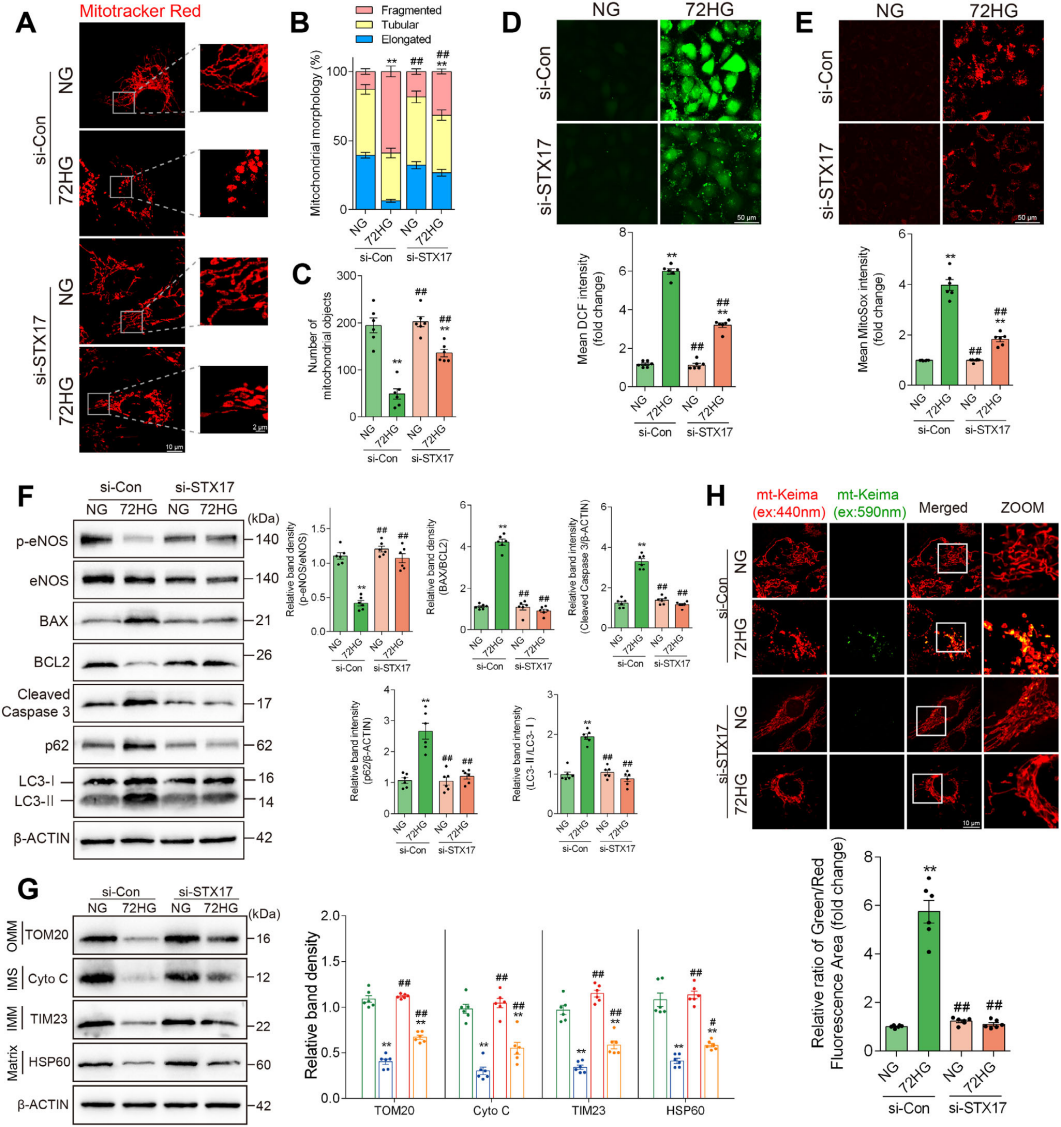
Silencing STX17 alleviated high‐glucose‐induced vascular endothelial injury. HUVECs were transfected with siRNA against control (si‐Con) or STX17 (si‐STX17) and then exposed to normal glucose (NG group) and 72 h of high glucose (72HG group). A) HUVECs were stained with MitoTracker Red. Mitochondrial length B) and total number of fragmented, tubular, and elongated mitochondrial structures C) were quantitatively analyzed in each group. *n* = 6. D,E) ROS and mitochondrial ROS production were measured by DCF and MitoSOX staining, respectively. *n* = 6. F) Representative Western blot images and corresponding relative intensities of BAX, BCL2, cleaved caspase 3, p‐eNOS, eNOS, LC3, and p62 bands in HUVECs. n = 6. G) Representative Western blot images and corresponding relative intensities of TOM20, Cyto C, TIM23, and HSP60, representing proteins from the OMM, IMS, IMM, and matrix. *n* = 6. H) HUVECs stably expressing mitochondrial‐targeted Keima (mt‐Keima) were imaged by confocal microscopy with excitation at 440 nm (red) and 590 nm (green). Scale bar = 10 µm. n = 6. Data are expressed as mean ± SEM. ^*^ *p* < 0.05; ^**^ *p* < 0.01 versus NG + si‐Con group; ^#^ *p* < 0.05, ^##^ *p* < 0.01 versus 72HG + si‐Con group.

To investigate the involvement of STX17‐mediated mitophagy in vascular endothelial injury following high‐glucose exposure, we treated HUVECs with high glucose for 72 h, followed by transfection with STX17‐targeting siRNA (si‐STX17) to block STX17‐mediated mitophagy (Figure S4A,B, Supporting Information). Mitochondrial fragmentation and loss induced by 72 h of high‐glucose exposure were significantly reduced by si‐STX17 transfection (**Figure** **6**A–C). Consistent with these morphological changes, Western blot analysis revealed a significant increase in overall mitochondrial proteins in si‐STX17‐transfected HUVECs following 72 h high‐glucose exposure, including TOM20 (outer mitochondrial membrane protein), Cyto C (intermembrane space protein), TIM23 (inner membrane mitochondrial protein) and HSP60 (mitochondrial matrix protein), compared with si‐Control transfected HUVECs following 72 h high‐glucose exposure (Figure 6G). Then, the role of STX17‐mediated mitophagy in apoptosis was investigated. Notably, transfection with si‐STX17 reduced apoptosis induced by 72 h of high‐glucose exposure, indicated by decreased cleaved caspase 3 expression and a reduced Bax/Bcl‐2 ratio (Figure 6F). Moreover, si‐STX17 increased eNOS phosphorylation, improving endothelial function (Figure 6F). DCF and MitoSOX staining showed that blocking STX17‐mediated mitophagy suppressed ROS and mitochondrial ROS overproduction (Figure 6D,E). The elevated overall mitophagy was reduced by si‐STX17 following 72 h of high‐glucose exposure in HUVECs, indicating that STX17‐mediated mitophagy is the predominant mitophagy pathway activated by long‐term high‐glucose exposure (Figure 6F,H). These findings suggest long‐term high‐glucose exposure induces STX17‐mediated mitophagy, potentially leading to vascular endothelial injury. Silencing STX17 protects against vascular endothelial injury caused by high glucose.

### 2.5 Fis1 Is Involved in the Switch Between Parkin‐Mediated Mitophagy and STX17‐Mediated Mitophagy

**Figure 7:**
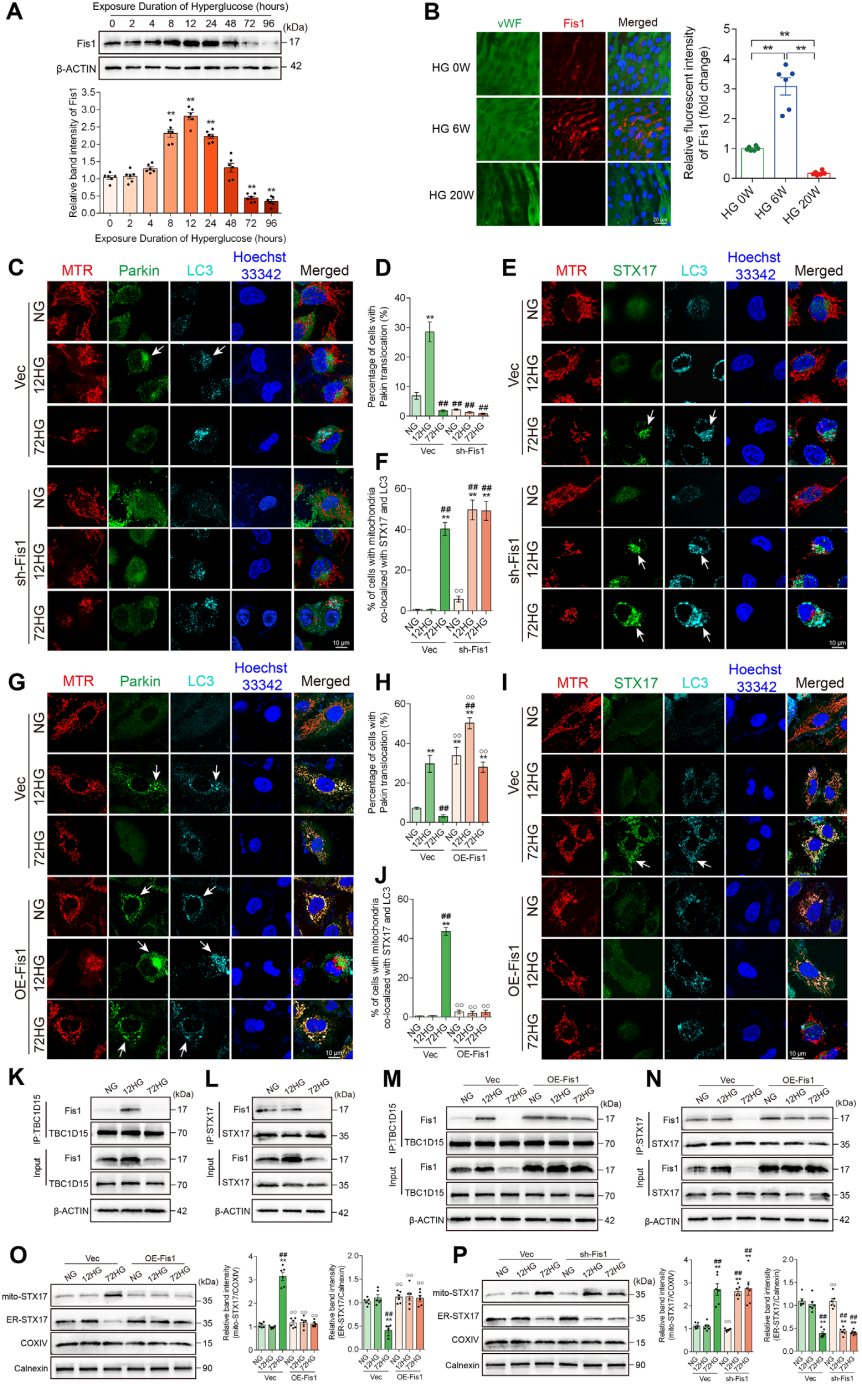
Fis1 modulates Parkin‐mediated mitophagy and STX17‐mediated mitophagy in high‐glucose conditions. A) Representative Western blot bands and relative band intensities showing temporal changes in Fis1 expression following high‐glucose exposure. *n* = 6. B) Male ApoE^−/−^ mice (6 weeks old) were injected with STZ to induce hyperglycemia. Mouse aortas were collected at 0 weeks (HG 0 W), 6 weeks (HG 6 W), and 20 weeks (HG 20 W) following hyperglycemia exposure. En face immunofluorescence staining of the mice abdominal aorta with immunofluorescent density analysis. Red, green, and blue represent Fis1, VWF (an endothelial cell marker) and DAPI (a nuclear marker). Scale bar = 20 µm. *n* = 6. C–J, M–P) HUVEC cell lines with Fis1 overexpression (OE‐Fis1) and silencing (sh‐Fis1) were constructed using lentivirus‐mediated transduction and then exposed to normal glucose (NG group), 12 h of high glucose (12HG group), and 72 h of high glucose (72HG group). C,D) Fis1‐silencing HUVECs were stained with MitoTracker (red) and analyzed by immunofluorescence microscopy for Parkin (green) and LC3 (cyan). Nuclei were stained with Hoechst (blue). *n* = 6. E,F) Fis1‐silencing HUVECs were labeled with MitoTracker (red) and analyzed using immunofluorescence microscopy to detect STX17 (green) and LC3 (cyan), with nuclei stained with Hoechst (blue). *n* = 6. G,H) Fis1‐overexpression HUVECs were stained with MitoTracker (red) and analyzed by immunofluorescence microscopy for Parkin (green) and LC3 (cyan). Nuclei were stained with Hoechst (blue). *n* = 6. I,J) Fis1‐overexpression HUVECs were labeled with MitoTracker (red) and analyzed using immunofluorescence microscopy to detect STX17 (green) and LC3 (cyan), with nuclei stained with Hoechst (blue). Scale bar = 10 µm. *n* = 6. K–L) Co‐immunoprecipitation analysis of the interaction of K) Fis1 and TBC1D15, L) Fis1 and STX17 in HUVECs exposed to normal glucose (NG group), 12 h of high glucose (12HG group), and 72 h of high glucose (72HG group). *n* = 3. M,N) Co‐immunoprecipitation analysis of M) Fis1 and TBC1D15 interaction, N) Fis1 and STX17 in Fis1‐overexpression HUVECs. *n* = 3. O,P) Representative Western blot bands and relative band intensities showing STX17 expression in mitochondria and the endoplasmic reticulum (ER) of O) Fis1‐overexpression and P) Fis1‐silencing HUVECs, with COXIV used as a mitochondrial marker and Calnexin as an ER marker. *n* = 6. Data are expressed as mean ± SEM. A) ^*^ *p* < 0.05; ^**^ *p* < 0.01 versus 0‐h high‐glucose exposure group. B) ^*^ *p* < 0.05; ^**^ *p* < 0.01. C‐P) ^*^ *p* < 0.05; ^**^ *p* < 0.01 versus NG + Vec group; ^#^ *p* < 0.05, ^##^ *p* < 0.01 versus 12HG + Vec group; ^°^ *p* < 0.05, ^°°^ *p* < 0.01 versus 72HG + Vec group. Vec, vector.

Our findings indicate that inhibition of Parkin‐mediated mitophagy, combined with activation of STX17‐mediated mitophagy in endothelial cells exposed to long‐term high‐glucose, might lead to endothelial injury. Given that Fis1‐recruited TBC1D15 regulates Parkin‐mediated mitophagy,^[^ ^22^ ^]^ while Fis1 loss promotes STX17‐initiated mitophagy,^[^ ^19^ ^]^ the involvement of Fis1 in modulating both Parkin‐mediated and STX17‐mediated mitophagy following high‐glucose exposure was explored. The expression of Fis1 was continuously measured in HUVECs exposed to high glucose. Notably, Fis1 expression initially increased and decreased over time (**Figure** **7**A). Similarly, en‐face fluorescence staining of mouse vasculature showed a significant upregulation of Fis1 in the HG 6 W group, followed by a decrease in the HG 20 W group (Figure 7B). The co‐immunoprecipitation analysis indicated that the interaction of Fis1 with TBC1D15 was significantly enhanced in the 12HG group compared with the NG group. As anticipated, long‐term high‐glucose exposure reduced the interaction of Fis1 with TBC1D15 and the combination of Fis1‐STX17 in HUVECs (Figure 7K,L).

To investigate the involvement of Fis1 in modulating Parkin‐mediated and STX17‐mediated mitophagy under high‐glucose conditions, we constructed lentivirus‐mediated stable HUVEC cell lines with Fis1 overexpression and silencing (Figure S5A,B, Supporting Information). Fis1 silencing inhibited Parkin‐mediated mitophagy (Figure 7C,D) and promoted STX17‐mediated mitophagy (Figure 7E,F) in the 12‐hour HG‐treated groups. Further Western blot analysis of STX17 confirmed that silencing of Fis1 in the 12 h of HG‐treated groups significantly stimulated the translocation of STX17 from the ER to mitochondria (Figure 7O,P) compared with 12 h of HG‐treated VEC groups, accelerating the initiation of STX17‐mediated mitophagy. In contrast, Fis1 overexpression in 72‐h HG‐treated groups significantly restored Parkin‐mediated mitophagy (Figure 7G,H). They prevented the initiation of STX17‐mediated mitophagy (Figure 7I,J) compared with 72‐h HG‐treated VEC groups. Moreover, silencing Fis1 disrupted the association between Fis1 and STX17, starting at 12 h of high‐glucose exposure in HUVECs. Conversely, Fis1 overexpression maintained the interaction between Fis1 and TBC1D15 and the association between Fis1 and STX17, even after 72 h of high‐glucose exposure (Figure 7M,N). These findings suggest that short‐term high‐glucose exposure upregulates Fis1 expression, enhancing its interaction with TBC1D15 and promoting Parkin‐mediated mitophagy. In contrast, long‐term high‐glucose exposure reduces Fis1 expression, inhibiting Parkin‐mediated mitophagy and promoting the translocation of STX17 from the ER to mitochondria, shifting mitophagy to an STX17‐mediated pathway. Furthermore, silencing Fis1 accelerates the switching from Parkin‐mediated to STX17‐mediated mitophagy. Overexpression of Fis1 prevents the switching from Parkin‐mediated to STX17‐mediated mitophagy following long‐term high‐glucose exposure.

### 2.6 Overexpression of Fis1 Alleviated High‐Glucose‐Induced Vascular Endothelial Injury

**Figure 8:**
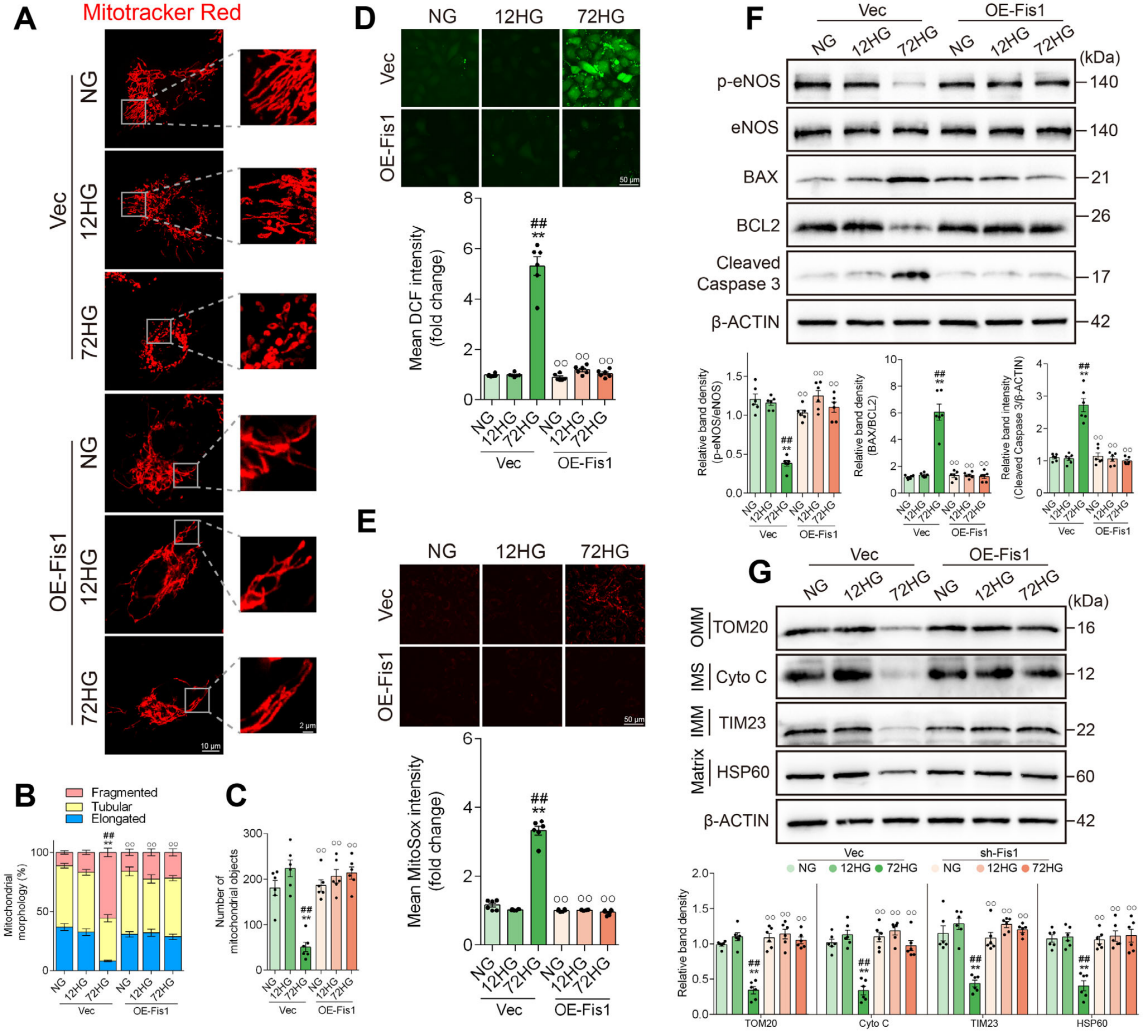
Fis1 overexpression alleviated high‐glucose‐induced vascular endothelial injury in HUVECs exposed to long‐term high‐glucose. HUVEC cell lines with Fis1 overexpression (OE‐Fis1) were constructed using lentivirus‐mediated transduction and then exposed to normal glucose (NG group), 12 h of high glucose (12HG group), and 72 h of high glucose (72HG group). A) HUVECs were stained with MitoTracker Red. Mitochondrial length B) and total number of fragmented, tubular, and elongated mitochondrial structures C) were quantitatively analyzed in each group. Scale bar = 10 µm. *n* = 6. D‐E) ROS and mitochondrial ROS production were measured by DCF and MitoSOX staining, respectively. Scale bar = 50 µm. *n* = 6. F) Representative Western blot images and corresponding relative intensities of BAX, BCL2, cleaved caspase 3, p‐eNOS and eNOS bands in HUVECs. *n* = 6. G) Representative Western blot images and corresponding relative intensities of TOM20, Cyto C, TIM23, and HSP60, representing proteins from the OMM, IMS, IMM, and matrix. *n* = 6. Data are expressed as mean ± SEM. ^*^ *p* < 0.05; ^**^ *p* < 0.01 versus NG + Vec group; ^#^ *p* < 0.05, ^##^ *p* < 0.01 versus 12HG + Vec group; ^°^ *p* < 0.05, ^°°^ *p* < 0.01 versus 72HG + Vec group. Vec, vector.

Mitochondrial fragmentation and a reduction in overall mitochondrial protein levels were observed in HUVECs treated with 72 h of high glucose compared to the NG group; both were reversed by Fis1 overexpression (**Figure** **8**A–C,G). Furthermore, Western blot analysis demonstrated that Fis1 overexpression alleviated ROS and mitochondrial ROS production (Figure 8D,E) and apoptosis (Figure 8F) caused by 72 h of high‐glucose exposure, as evidenced by decreased ROS and mitochondrial ROS production, a reduced BAX/BCL2 ratio, and lower cleaved caspase 3 expression. Additionally, Fis1 overexpression significantly increased eNOS phosphorylation following 72 h of high‐glucose exposure, indicating the role of Fis1 in enhancing endothelial function (Figure 8F). These observations suggested that Fis1 overexpression reduced ROS production and apoptosis and improved endothelial dysfunction in vascular endothelial cells exposed to long‐term high glucose.

### 2.7 EC‐Specific Fis1 Silencing Exacerbated Hyperglycemia‐Induced Vascular Endothelial Injury

**Figure 9:**
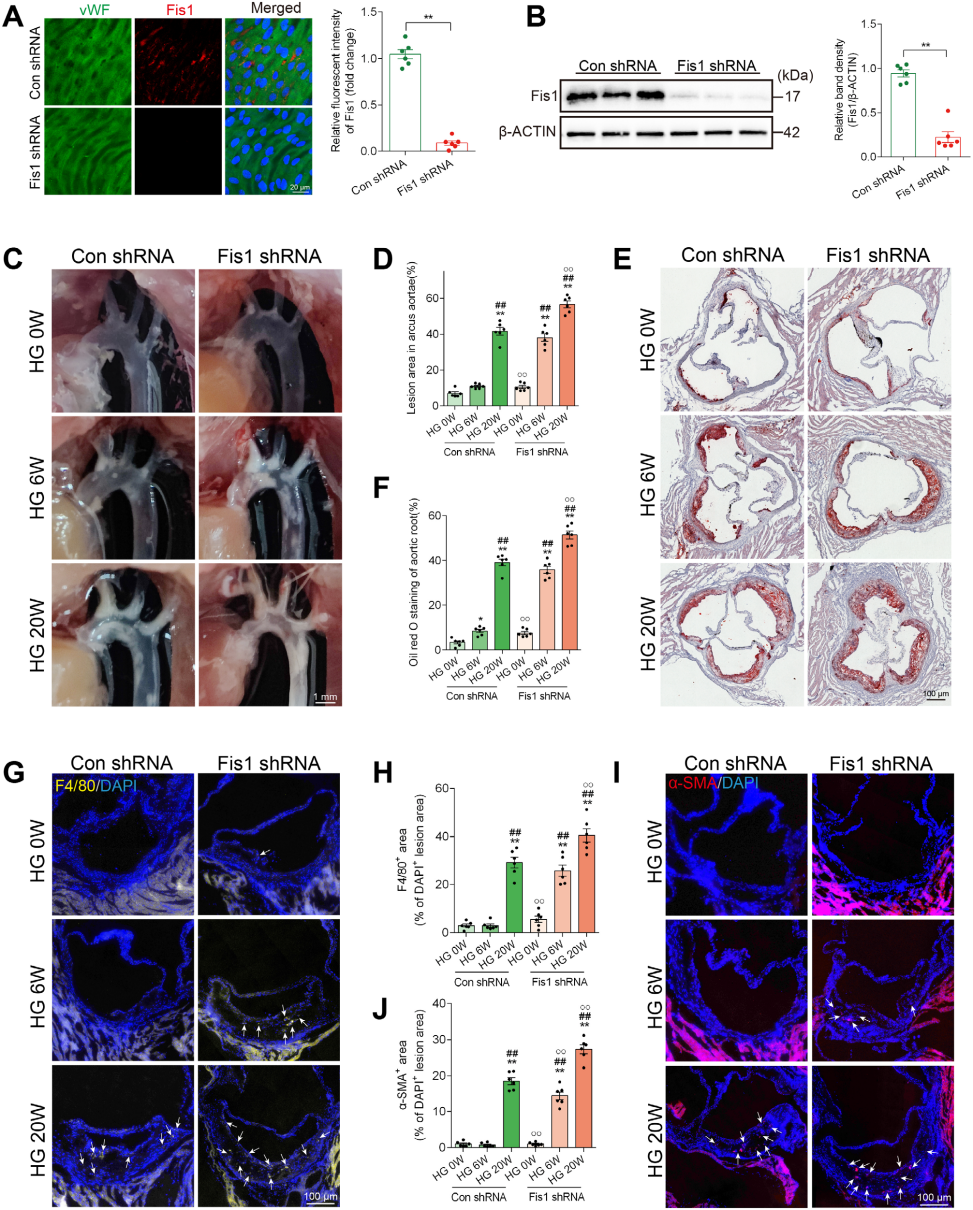
EC‐specific Fis1 silence exacerbated hyperglycemia‐induced vascular endothelial injury in STZ‐induced diabetic mice. ApoE^−/−^ mice were injected via the tail vein with recombinant adeno‐associated virus 9 (AAV9) containing Fis1 shRNA or control shRNA, driven by the endothelial cell‐specific Tie promoter. The mice were then exposed to hyperglycemia for 0 weeks (HG 0 W), 6 weeks (HG 6 W), or 20 weeks (HG 20 W). A) En face immunofluorescence staining of the abdominal aorta with immunofluorescent density analysis from the Con shRNA and Fis1 shRNA groups. Red, green, and blue represent Fis1, VWF (an endothelial cell marker) and DAPI (a nuclear marker). Scale bar = 20 µm. *n* = 6. B) Representative Western blot images and corresponding relative intensities of Fis1 expression in the aorta from Con shRNA and Fis1 shRNA groups. *n* = 6. C,D) Representative in situ images of atherosclerotic plaque in the aortic arch (C) with statistical analysis (D), expressed as % of total arcus aortae area. Scale bar = 1 mm. *n* = 6. E,F) Oil Red O staining of cross‐sections at the aortic root (E) with statistical analysis (F), expressed as % of the total aortic root area. Scale bar = 100 µm. *n* = 6. G‐H) Representative images (G) and statistical analysis (H) of macrophage infiltration (F4/80) immunostaining in aortic root plaque, expressed as % of DAPI^+^ lesion area. Scale bar = 100 µm. *n* = 6. I,J) Representative images (I) and statistical analysis (J) of α‐smooth muscle actin (α‐SMA) immunostaining in aortic root plaque, expressed as % of DAPI^+^ lesion area. Scale bar = 100 µm. n = 6. Data are expressed as mean ± SEM. A,B) ^*^ *p* < 0.05; ^**^ *p* < 0.01. C–J) ^*^ *p* < 0.05; ^**^ *p* < 0.01 versus HG 0 W + Con shRNA group; ^#^ *p* < 0.05, ^##^ *p* < 0.01 versus HG 6 W + Con shRNA group; °p < 0.05, °°p < 0.01 versus HG 20 W + Con shRNA group.

To investigate the role of Fis1 in hyperglycemia‐induced vascular endothelial injury, ApoE^−/−^ mice were injected via the tail vein with AAV9 (recombinant adeno‐associated virus 9) containing Fis1 shRNA, driven by the endothelial cell‐specific Tie promoter. Hyperglycemia was then induced in these mice by injecting STZ. Fis1 expression in the aortic intima of the mice injected with Fis1 shRNA was markedly reduced compared to mice injected with control shRNA, as confirmed by western blot (**Figure** **9**B) and vascular en face fluorescence staining (Figure 9A). In situ images (Figure 9C,D) and Oil Red O staining (Figure 9E,F) of mouse aortas showed that Fis1 silencing in ECs accelerated and aggravated atherosclerosis lesions compared with control shRNA mice. Macrophage content (Figure 9G,H) and the smooth muscle cell content (Figure 9I,J) in atherosclerotic lesions were higher in mice transduced with Fis1 shRNA starting from 6 weeks of hyperglycemia exposure and further aggravated following 20 weeks of hyperglycemia exposure, compared to the control shRNA group.

Consistent with in vivo data, oxidative stress (Figure S5D,E, Supporting Information) and cell apoptosis (Figure S5F, Supporting Information) were higher in 12‐h HG‐treated Fis1‐silenced HUVECs than in 12‐h HG‐treated control cells. Apoptosis and oxidative stress were further exacerbated in 72 h HG‐treated Fis1‐silenced HUVECs, as reflected by increased BAX/BCL2 ratio, elevated levels of cleaved caspase 3, and heightened levels of ROS and mitochondrial ROS production. Additionally, eNOS phosphorylation was markedly decreased in Fis1‐silencing HUVECs treated with high glucose for 12 h compared to the control group (Figure S5F, Supporting Information). Fis1 silencing also significantly increased mitochondrial fragmentation in 12 h of HG‐treated HUVECs compared with 12 h of HG‐treated controls (Figure S5C, Supporting Information). Consistent with these morphological changes, Western blot analysis showed a significant reduction in overall mitochondrial proteins in 12‐h HG‐treated Fis1‐silenced HUVECs compared with controls (Figure S5G, Supporting Information). Both in vivo and in vitro experiments suggest that Fis1 may play a critical role in hyperglycemia‐induced vascular endothelial injury.

## 3 Discussion

**Figure 10:**
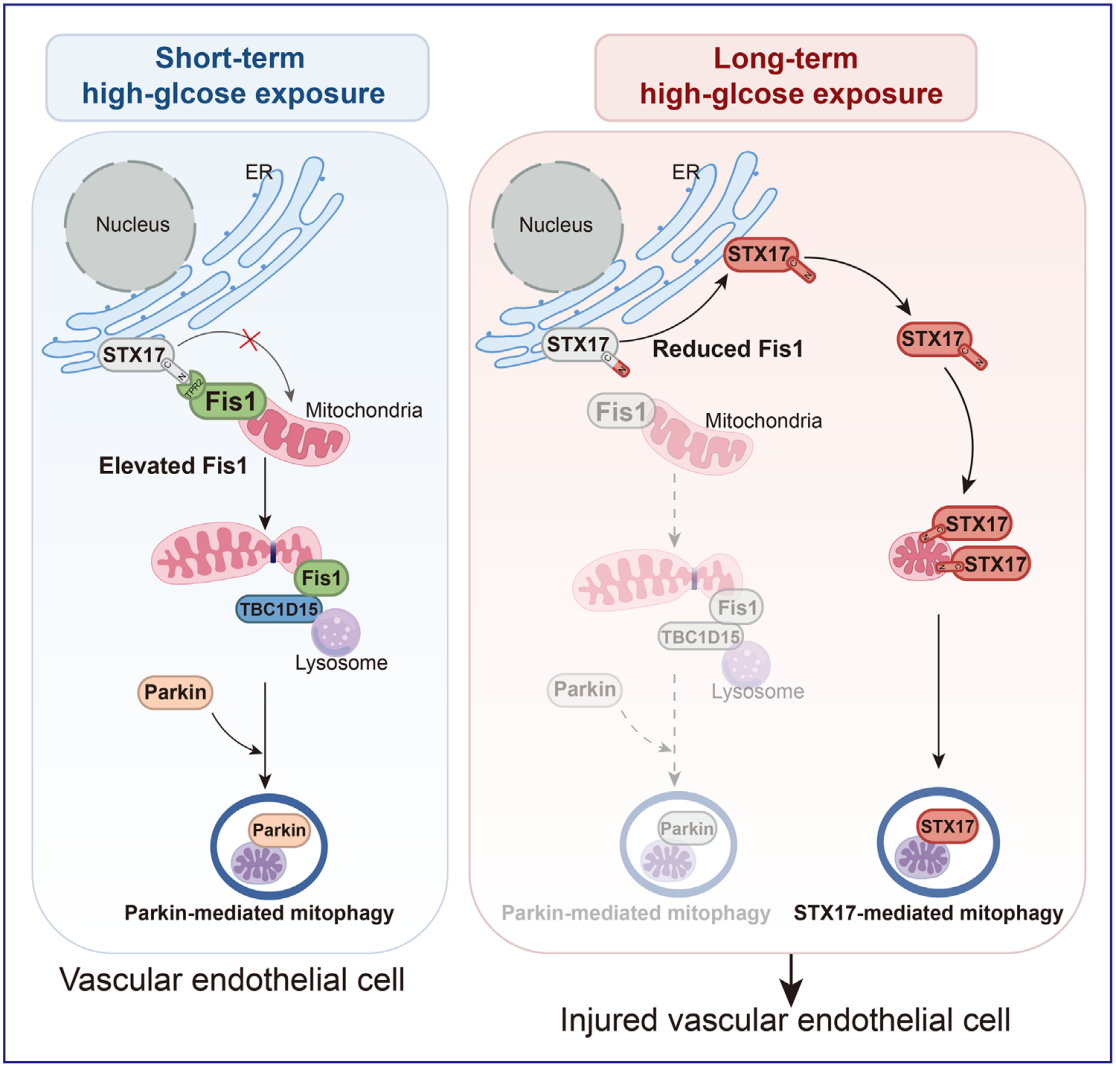
The model depicts the distinct mitophagy pathways activated following short‐term and long‐term high‐glucose exposure in vascular endothelial cells. Short‐term high‐glucose exposure (left) elevates Fis1 expression and enhances the interaction between Fis1 and TBC1D15, promoting Parkin‐mediated mitophagy. Meanwhile, Fis1 inhibits the exposure of STX17's N‐terminus and prevents its translocation from the ER to the mitochondria. In contrast, long‐term high‐glucose exposure (right) reduces Fis1 expression, inhibiting Parkin‐mediated mitophagy. The reduction in Fis1 expression exposes the N‐terminus of STX17, promoting STX17 translocation from the ER to the mitochondria and leading to its accumulation in the mitochondria. This process initiates STX17‐mediated mitophagy, ultimately leading to vascular endothelial injury.

In the present study, we demonstrated that mitophagy switching drives vascular injury following prolonged high‐glucose exposure. Long‐term high‐glucose exposure reduces Fis1 expression, inhibiting Parkin‐mediated mitophagy and triggering the translocation of STX17 from the endoplasmic reticulum to mitochondria in vascular endothelial cells. This switching from Parkin‐mediated to STX17‐mediated mitophagy drives endothelial dysfunction and vascular injury (**Figure** **10**). Our study has identified mitophagy switching as an important factor in vascular endothelial injury, offering potential insights into therapeutic strategies for diabetic cardiovascular complications.

Previous clinical studies suggest that there remains a continuing increased risk of death from diabetic cardiovascular complications despite improvements in glycemic control, a phenomenon known as “metabolic memory”.^[^ ^3^, ^5^, ^23^ ^]^ However, the critical mechanisms by which hyperglycemia triggers persistent vascular injury have not yet been elucidated fully. Our previous results showed that mitochondrial dynamics undergoes three stages during high‐glucose‐induced vascular injury: the compensation stage, balance shifting stage, and decompensation stage.^[^ ^24^ ^]^ During this progression, Fis1 expression significantly increased at the initial compensation and balance‐shifting stages. Once Fis1 expression decreased below normal levels and maintained at a low level, this change signified the transition into the decompensation stage. In the present study, “short‐term” and “long‐term” high‐glucose exposure were defined based on the changes in Fis1 expression levels. During the Fis1 upregulation stage, persistent vascular damage had not yet developed, which was defined as short‐term high‐glucose exposure. Both 12 h of high‐glucose exposure in vitro and 6 weeks of hyperglycemia exposure in vivo showed no endothelial damage, which was classified as short‐term high‐glucose exposure. As Fis1 expression declined below normal levels, vascular endothelial cells underwent persistent apoptosis, defining this stage as long‐term high‐glucose exposure. Both 72 h of high‐glucose exposure in vitro and 20 weeks of hyperglycemia exposure in vivo led to endothelial cell apoptosis, which was classified as long‐term high‐glucose exposure. In diabetic patients, “short‐term” and “long‐term” high‐glucose exposure may also be defined based on changes in Fis1 expression levels. Vita et al. reported that in diabetic patients ≈50 years old, vascular function remained unchanged, while their Fis1 expression was increased in endothelial cells freshly isolated from these patients compared to controls.^[^ ^25^ ^]^ This finding is consistent with our in vivo and in vitro results of short‐term high‐glucose exposure. The reduced Fis1 expression in endothelial cells of diabetic patients has not yet been reported. However, Houzelle et al. reported that Fis1 protein levels were significantly decreased in the muscles of individuals with type 2 diabetes compared to athletes, and these diabetic patients exhibited impaired insulin sensitivity.^[^ ^26^ ^]^ We speculate that their vascular endothelial function may also be impaired at this stage, which merits further investigation in future clinical studies. Although our in vivo and in vitro experiments do not fully reflect clinical conditions, future clinical studies may define “short‐term” and “long‐term” high‐glucose exposure based on changes in Fis1 levels in endothelial cells of diabetic patients.

To investigate the mechanisms underlying hyperglycemia‐induced vascular injury, we conducted a 4D‐label‐free quantitative proteomic analysis of whole aortas from three groups of diabetic ApoE^−/−^ mice: HG 0 W, HG 6 W, and HG 20W. Compared to HG 0 W, the differentially expressed proteins in the HG 6 W group differed from those in the HG 20 W group, but both were enriched in “mitochondrion” and “lysosome” pathways, which suggest that mitochondrial and autophagy‐related mechanisms may play crucial roles in hyperglycemia‐induced vascular injury. Due to limitations in observing mitochondria and lysosomes in vivo, mitochondrial‐lysosome interactions, co‐localization of LC3 and TOM20, and mt‐Keima measurements were observed in HUVECs. These findings demonstrate that mitophagy is activated following both short‐term and long‐term high‐glucose exposure. Classic Parkin‐mediated mitophagy is activated following short‐term high‐glucose exposure, whereas long‐term exposure suppresses it. Interestingly, silencing Parkin did not reduce the overall mitophagy levels induced by long‐term high‐glucose exposure, suggesting that alternative mitophagy pathways are activated under these conditions. Long‐term high‐glucose exposure triggered a Parkin‐independent, membrane depolarization‐independent mitophagy pathway, as evidenced by tracking daughter mitochondria resulting from mitochondrial fission and observing their subsequent outcomes. Further quantitative proteomic analysis and experiments demonstrated that long‐term high‐glucose exposure reduces Fis1 expression, thereby inducing STX17‐mediated mitophagy. This process promotes the translocation of STX17 from the ER to the mitochondria, ultimately leading to excessive mitochondrial clearance and cell apoptosis. In summary, long‐term exposure triggers a switch in mitophagy from Parkin‐mediated to STX17‐mediated mitophagy. Additionally, we found that Fis1 plays a key role in this switching process.

ECs are essential in the onset and progression of vascular complications in diabetes.^[^ ^27^ ^]^ Although mitochondria comprise just 2% to 6% of the cytoplasmic volume in ECs and have a limited energy supply,^[^ ^28^ ^]^ they are considered vital signaling hubs in responding to oxidative stress, calcium buffering, and apoptosis.^[^ ^29^ ^]^ Mitophagy, as a regulatory mechanism for maintaining mitochondrial homeostasis, selectively removes damaged and depolarized mitochondria, thereby protecting ECs from hyperglycemia‐induced injury.^[^ ^30^ ^]^ Several drugs have been found to promote mitophagy, mitigating high‐glucose‐induced EC damage.^[^ ^31^ ^]^ However, it is worth noting that mitophagy in ECs is not always beneficial and may also result in cell damage^[^ ^32^ ^]^ and death.^[^ ^33^ ^]^ For instance, vitamin D and salvianolic acid B have been shown to inhibit mitophagy, protecting ECs from apoptosis.^[^ ^13^ ^]^ Additionally, dynamic changes in mitophagy occur during disease progression. Increased mitophagy is an early response to mitochondrial damage, but stalled mitophagy can exacerbate symptoms as the condition progresses.^[^ ^34^ ^]^ Acute lipid exposure promotes cellular autophagy as a protective mechanism, and prolonged lipid stimulation inhibits autophagy by disrupting autophagosome‐lysosome fusion.^[^ ^35^ ^]^ Thus, mitophagy acts as a double‐edged sword for endothelial cells, as it can cause different results depending on disease progression. In this study, short‐term high‐glucose exposure activated Parkin‐mediated mitophagy, while long‐term high‐glucose exposure activated STX17‐mediated mitophagy, leading to different outcomes. Parkin‐mediated mitophagy is highly selective, targeting damaged mitochondria with membrane depolarization. This pathway efficiently eliminates damaged mitochondria, protecting cells from oxidative stress and apoptosis.^[^ ^14^, ^15^ ^]^ High‐glucose‐induced vascular endothelial injury was accelerated and aggravated by si‐Parkin, suggesting that Parkin‐mediated mitophagy plays a critical role in protecting against endothelial injury during short‐term high‐glucose exposure. In contrast, STX17‐mediated mitophagy represents a Parkin‐independent pathway targeting damaged and healthy mitochondria.^[^ ^36^ ^]^ In this pathway, the loss of Fis1 causes STX17 to translocate from the endoplasmic reticulum (ER) to the mitochondria, where it recruits essential autophagic components such as ATG14, facilitating mitophagosome formation. This process triggers non‐selective mitophagy, potentially leading to excessive mitochondrial clearance. Xian et al. reported that HeLa cells exhibited a significantly increased population of dead cells with permeable plasma membranes during STX17‐mediated mitophagy, substantiating the role of STX17‐mediated mitophagy in cell death.^[^ ^19^ ^]^ Our results indicate that endothelial cells exposed to long‐term high glucose may activate STX17‐mediated mitophagy, leading to excessive mitochondrial degradation, ultimately resulting in apoptosis. Silencing of STX17 alleviated high‐glucose‐induced endothelial cell apoptosis and vascular endothelial injury, highlighting the detrimental role of STX17‐mediated mitophagy in maintaining vascular endothelial homeostasis. As STX17‐mediated mitophagy is a relatively newly discovered pathway, the specific mechanism by which STX17‐mediated mitophagy causes cell injury or dysfunction remains to be further studied. Based on these findings, this work suggests a potential therapeutic strategy: selectively activating Parkin‐mediated mitophagy while inhibiting STX17‐mediated mitophagy to protect ECs from high‐glucose‐induced injury rather than focusing solely on the activation or inhibition of overall mitophagy levels.

Mitochondrial fission protein 1 (Fis1), located on the outer mitochondrial membrane, plays a crucial role in the process of mitochondrial fission.^[^ ^37^ ^]^ Growing evidence indicates that Fis1 also has a prominent role in mitophagy.^[^ ^38^ ^]^ While the exact mechanistic details of Fis1 in mitophagy remain unclear. Recently, A groundbreaking discovery indicated that mitochondrial peripheral fission (occurring within 25% of the tip) divides metabolically healthy organelles from unhealthy ones, with the damaged daughter mitochondrion subsequently removed through mitophagy, and Fis1 is essential for regulating peripheral fission.^[^ ^15^, ^20^ ^]^ Fis1 facilitates peripheral fission to promote cell survival under stress by interacting with TBC1D15, a GTPase‐activating protein that modulates RAB7 GTP hydrolysis.^[^ ^22^, ^39^ ^]^ This peripheral fission process is crucial for facilitating Parkin‐mediated mitophagy, a pathway critical for the selective removal of damaged mitochondria and maintaining mitochondrial quality control. Peripheral mitochondrial fission has risen under different cellular stress conditions, including oxidative stress and nutrient deprivation, highlighting its role in cellular adaptation and survival.^[^ ^15^ ^]^ However, the role of peripheral fission in mitochondrial dynamics and its potential contribution to mitophagy in high‐glucose‐induced endothelial dysfunction still need to be explored. Our study revealed that short‐term high‐glucose exposure enhanced the Fis1‐TBC1D15 interaction, promoting peripheral mitochondrial fission and activating Parkin‐mediated mitophagy as a protective mechanism to mitigate the acute mitochondrial stress induced by hyperglycemia. However, long‐term high‐glucose exposure suppressed this interaction, inhibiting Parkin‐mediated mitophagy and shifting toward STX17‐mediated mitophagy. Fis1‐mediated STX17 mitophagy represents a distinct, Parkin‐independent mitophagy pathway.^[^ ^18^, ^19^ ^]^ In this pathway, the loss of Fis1 causes STX17 to translocate from the endoplasmic reticulum (ER) to mitochondria, where it recruits essential autophagic components such as ATG14, facilitating the formation of mitophagosomes.^[^ ^19^, ^40^ ^]^ This process triggers non‐selective mitophagy, potentially leading to excessive mitochondrial clearance.^[^ ^19^ ^]^ However, the role of STX17‐initiated mitophagy in various physiological states remains unclear. Our results indicated long‐term high‐glucose exposure induced STX17‐mediated mitophagy, leading to excessive mitochondrial clearance and cell apoptosis. Silencing STX17 alleviated high‐glucose‐induced vascular endothelial injury. Furthermore, silencing Fis1 inhibited Parkin‐mediated mitophagy while inducing STX17‐mediated mitophagy, which led to endothelial damage following short‐term high‐glucose exposure in HUVECs. In ApoE^−/−^ mice, EC‐specific Fis1 silencing exacerbated vascular endothelial injury following long‐term high‐glucose exposure. These findings suggest that Fis1 is essential in hyperglycemia‐induced vascular injury, indicating its potential as a therapeutic target. While Fis1 regulation has shown benefits in various disease models, our study revealed that overexpression of Fis1 during long‐term high‐glucose exposure reduced endothelial cell injury. These findings suggest that enhancing Fis1 expression might be beneficial to restore mitophagy balance and reduce cellular damage following long‐term high‐glucose exposure.

We have demonstrated that high‐glucose exposure triggers switching from Parkin‐mediated to STX17‐mediated mitophagy; whether other pathogenic factors can initiate this switching remains unclear. Additionally, the precise regulatory mechanisms controlling mitophagy switching require further investigation. In summary, our findings reveal that mitophagy switching from Parkin‐mediated to STX17‐mediated mitophagy drives vascular endothelial injury, providing valuable perspectives on therapeutic strategies for diabetic cardiovascular complications. Mitophagy switching may serve as an indicator of disease progression in high‐glucose‐induced vascular injury in diabetic patients.

## 4 Experimental Section

### Animals and Treatments

The apoE knockout mice (C57BL/6 background, 5 weeks old) were obtained from Cyagen Biosciences. Six‐week‐old male mice were given daily intraperitoneal injections of streptozotocin (STZ) at 55 mg kg^−1^ for five consecutive days to induce hyperglycemia. One week after the last injection, mice with fasting blood glucose levels of ≥16.7 mmol L^−1^ were chosen for the study. The hyperglycemic mice were separated into two groups: one group was fed for 6 weeks (HG 6 W group) and the second group for 20 weeks (HG 20 W group). Aortas were collected from both groups at the respective time points. Control mice (HG 0 W group) were administered vehicle injections of 0.1 mol L^−1^ citrate‐phosphate buffer (pH 4.5). They had aortas collected one week after the injections.

Genomeditech Biotechnology (Shanghai, China) constructed the adenoviruses. To specifically knock down Fis1 protein expression in the endothelium of ApoE^−/−^ mice, adeno‐associated virus serotype 9 (AAV9) carrying Fis1 shRNA driven by the endothelial‐specific Tie2 promoter was used. The Fis1 shRNA targeting sequence was 5′‐CCTGATTGATAAGGCCATGAA‐3′, and the control shRNA sequence was 5′‐TTCTCCGAACGTGTCACGT‐3′. Mice were injected via the tail vein with recombinant adeno‐associated virus 9 (AAV9) containing Fis1 shRNA (1.5 × 10¹¹ vector genomes per mouse) under the supervision of the Tie2 promoter. The mice were then divided into HG 0 W, HG 6 W, and HG 20 W groups as described above. Aortas were collected for further analysis. All mice had free access to standard chow and water and were fasted for 8 h before taking blood glucose measurements.

### Reagents and Antibodies

The reagents listed below were utilized: MitoTracker Red FM (ThermoFisher, M22425), LysoTracker Green (ThermoFisher, L7526), MitoSOX Red (ThermoFisher, M36008), DCF (ThermoFisher, D399), tetramethylrhodamine ethyl ester perchlorate (Sigma, 87917), DAPI (ThermoFisher, 62248), Hoechst 33342 (ThermoFisher, 62249), TUNEL Apoptosis Assay Kit (keygentec, KGA1406), Mitochondrial Isolation Kit (Invent, MP‐007), ER Enrichment Kit (Invent, ER‐036), The reagents for Western blotting were obtained from Yeasen (Shanghai, China).

The following Antibodies were used: F4/80 (Proteintech, 29414, 1:200), α‐SMA (Proteintech, 14395, 1:200), p62 (Cell Signaling Technology, 23214, 1:5000) (Proteintech, 18420, 1:3000), LC3 (Cell Signaling Technology, 3868, 1:4000) (Cell Signaling Technology, 83506, 1:1000), vWF (Proteintech, 66682, 1:500), BAX (Proteintech, 60267, 1:5000), BCL2 (Proteintech, 60178, 1:1000), Caspase 3 (Proteintech, 66470, 1:2000), eNOS (Cell Signaling Technology, 9572, 1:2000), Phospho‐eNOS (Ser1177) (Cell Signaling Technology, 9570, 1:1500), Beta Actin (Proteintech, 66009, 1:10000), Tom20 (Proteintech, 11802, 1:5000), Tim23 (Proteintech, 67535, 1:2000), Cyto C (Proteintech, 66264, 1:5000), HSP60 (Proteintech, 66041, 1:10000), Parkin (Abcam, ab15494, 1:2000), STX17 (Abcam, ab229646, 1:1000), Fis1 (Abcam, ab229969, 1:3000), TBC1D15 (Abcam, ab121396, 1:1000), 3‐NT (EMD Millipore, 06–284, 1:1000), 8‐OHdG (Abcam, ab62623, 1:1000), COXIV (Proteintech, 11242, 1:5000), Calnexin (Proteintech, 10427, 1:5000).

### 4D‐Label Free Proteomics Analysis

The aortas of mice in the HG 0 , HG 6 , and HG 20 W groups were separated and subjected to 4D‐label‐free quantitative proteomic analysis. The main experimental steps for this analysis involved protein extraction and digestion, LC‐MS/MS analysis, and protein identification and quantification, followed by bioinformatic analysis. MaxQuant 1.6.14 software was used for protein identification and quantitation. Differentially expressed protein sequences were first analyzed using NCBI BLAST+ to identify homologous sequences, then GO terms were mapped and annotated with Blast2GO. Proteins were also compared against the KEGG database (http://geneontology.org/) to obtain KEGG orthology IDs. The proteomic analysis was supported by APTBIO (Shanghai, China).

### Histological Analyses

Aortic roots from mice were sectioned and stained using Oil Red O (Solarbio, China) according to the manufacturer's instructions. Cryosections of mouse aortic roots were fixed and incubated with 5% BSA. Sections were incubated with F4/80 or α‐SMA primary antibodies. After three washes with PBS, fluorescently labeled secondary antibodies (ThermoFisher, A‐21245, 1:1000) were applied. DAPI was used to stain nuclei. Fluorescent images of the aortic root tissues were captured using the Olympus FV3000 microscope.

The fresh thoracic aortas from mice were fixed in 4% formaldehyde, dehydrated, and then embedded in paraffin. Immunohistochemical staining was performed to detect 3‐nitrotyrosine (3‐NT) and 8‐oxo‐29‐deoxyguanosine (8‐OHdG). Hematoxylin kits (Solarbio, China) were then used for counterstaining. A semi‐quantitative analysis based on staining intensity (0: no staining, 1: faint yellow, 2: light brown, 3: brown) and the proportion of positive cells (0: 0%–5%, 1: 6%–25%, 2: 26%–50%, 3: 51%–75%, 4: 76%–100%). Paraffin‐embedded sections of mouse abdominal aortas were dewaxed, hydrated, and blocked. The TUNEL Apoptosis Assay Kit assessed apoptosis following the manufacturer's instructions.

Mouse abdominal aortas were dissected, opened longitudinally, and fixed in 4% paraformaldehyde. The aortas were incubated with primary antibodies against LC3, P62, and Fis1. Following three washes in PBS, fluorescently labeled secondary antibodies were applied to the aortas.

### Cell Culture and Cell Transfection

HUVECs, obtained from the American Type Culture Collection (Manassas, VA, USA), were cultured in 10% FBS. HUVECs were exposed to a normal glucose medium (NG, 5.5 mm) for 0, 60, or 72 h and then shifted to a high‐glucose medium (HG, 33 mm). All cells were cultured for 72 h before harvesting, resulting in exposure to high glucose for 72, 12, or 0 h, respectively.

The small interfering RNAs (siRNAs) were synthesized by Invitrogen, with the following sequences: Control siRNA: 5′‐UUCUCCGAACGUGUCACGU‐3′; si‐Parkin: 5′‐GGAGUGCAGUGCCGUAUUU‐3′; and STX17 siRNA: 5′‐GAAAGTCCGAAAGGATGACCTAGTA‐3′. Using Lipofectamine 3000 reagent (Invitrogen) and following the manufacturer's protocol, HUVECs were transfected with the specified siRNAs.

### Lentiviral Transfection

Lentiviruses carrying Fis1 shRNA (sh‐Fis1; 5′‐AGGCCTTAAAGTACGTCCG‐3′) and control lentivirus (Vec; 5′‐TTCTTCGAACGTGTCACGT‐3′), lentiviruses for Fis1 overexpression (OE‐Fis1) and control (Vec) lentivirus were designed by Hanbio Biotechnology (Shanghai, China). HUVECs were seeded in 24‐well plates at 30%–50% confluence and infected with the specific lentiviruses at a multiplicity of infection (MOI) 30. Transfection efficiency was evaluated by fluorescence microscopy 72 h post‐infection (Olympus Corporation, Japan). Puromycin was added to select cells successfully virus‐infected.

### Mitophagy Analysis by Mt‐Keima Transfection

Mitophagy was monitored using mitochondria‐targeted Keima (mt‐Keima), which emits distinct colors in neutral and acidic environments. The mt‐Keima adenovirus was transfected following the manufacturer's instructions (Hanbio Biotechnology, Shanghai, China). Mt‐Keima signals were detected at 440 or 590 nm, where excitation at 440 nm represents mitochondria in the cytoplasm (red), and excitation at 590 nm indicates mitochondria in acidic lysosomes (green).

### Measurement of Oxidative Stress

ROS levels, indicative of oxidative stress in HUVECs, were assessed using DCF staining. MitoSox was used to evaluate mitochondrial ROS levels. Fluorescence from both ROS and mt‐ROS was detected using confocal microscopy (Olympus FV3000).

### Cell Immunofluorescence

Cells grown in confocal dishes were fixed. After blocking with 3% FBS/PBS, the cells were incubated with primary antibodies, followed by Alexa Fluor‐conjugated secondary antibodies. Nuclei were stained with Hoechst 33 342. Images were captured using a confocal microscope (Olympus FV3000), and co‐localization analysis was carried out with ImageJ software.

### Mitochondrial and Lysosome Imaging

Cells were treated with 500 nm Mitotracker Red for 5 min of incubation. The Opera Phenix High Content Screening System (PerkinElmer, Waltham, MA, USA) was used to acquire images, and mitochondrial quantification was conducted using Harmony software version 4.9. Live‐cell imaging was carried out in a chamber heated to 37 °C with 5% CO₂. Mitochondrial fissions were characterized as instances in live cells where a single mitochondrion divided into two independently moving daughter mitochondria, with the constriction site diameter measuring less than 180 nm. The mitochondrial skeleton was mapped to calculate the total length of the mitochondrion prior to fission. Peripheral fission was defined as occurring within 25% of the tip, while midzone fission occurred within the central 50% of the mitochondrion. Fission events were recorded as the number of fissions per 10 min in a single field of view.

Mitochondrial morphology was classified into three categories according to length: fragmented (less than 3 mm), tubular (3–8 mm), and elongated (greater than 8 mm). At least 200 mitochondria were analyzed for each experimental group.

Cells were treated with 500 nM MitoTracker Red and 500 nm LysoTracker Green. Images were taken using a confocal microscope (Olympus FV3000), and co‐localization analysis was performed with ImageJ software.

### Mitochondrial Membrane Potential

Cells received 500 nm TMRE treatment. The fluorescence intensity is positively correlated with membrane potential, where the fluorescence intensity is represented by color gradients.

### Subcellular Fractionation

Mitochondrial and ER fractions were isolated using the Mitochondrial Isolation Kit and ER Enrichment Kit (Invent Biotechnologies), following the manufacturer's protocols.

### Western Blotting and Co‐Immunoprecipitation

Cells were lysed in RIPA buffer. Protein samples were run on SDS‐PAGE and then incubated with primary antibodies. Following incubation with secondary antibodies, signals were detected using the ECL Kit and analyzed with ImageJ software.

Protein complexes were isolated using a co‐immunoprecipitation (Co‐IP) kit (Thermo Fisher Scientific). Cells were lysed in IP lysis buffer, and protein samples were incubated with the primary antibody. Pierce protein A/G beads were used to capture antigen‐antibody complexes. After being washed three times with washing buffer, the beads were used to elute the antigen‐antibody complexes, which were then analyzed by Western blot.

### Electron Microscopy

Cells were fixed with 2.5% glutaraldehyde, washed three times in PBS, and embedded in agarose. They were then post‐fixed in 1% osmium tetroxide and dehydrated through a graded ethanol series (30%, 50%, 70%, 80%, 95%, and 100%) for 20 min each, followed by two changes of acetone for 15 min. Next, the cells were infiltrated with EMBed 812 resin and polymerized overnight at 65 °C. Ultra‐thin sections (60–80 nm) were cut and collected on copper grids. The sections were stained with 2% uranyl acetate and 2.6% lead citrate before imaging with a transmission electron microscope (HITACHI, HT7800). Quantitative analysis of mitophagosome numbers was performed across randomly selected fields of view.

### Statistical Analysis

All data were presented as mean ± SEM, and the numbers of independent experiments were indicated. Student's t‐test was used to compare two groups, while one‐way ANOVA with Tukey's post‐test was applied to compare differences between multiple experimental groups. Data analysis was performed using GraphPad Prism software version 6.0 (SanDiego, CA, USA). Statistical significance was defined as *P* < 0.05.

### Ethics Approval Statement

The animal study protocol was approved by the Ethics Committee of Hainan Medical University (approval number: HYLL‐2023‐185).

## Conflict of Interest

The authors declare no conflict of interest.

## Author Contributions

A.L. and Y.Z. performed conceptualization. A.L., J.G., and S.W. performed methodology. R.W. and Y.J. performed validation. A.L. and R.W. performed formal analysis. A.L., J.G., S.W., and Y.J. performed investigation. X.L., H.D., H.L., and Y.Z. performed resources. X.L. and Y.Z. performed data curation. A.L. and Y.Z. wrote the original draft preparation. X.L. and Y.Z. wrote reviewed and edited the final manuscript. C.Y. and Z.C. performed visualization. H.D., H.L., and X.L. performed supervision. C.Y. and Z.C. performed project administration. X.L., H.D., and Y.Z. acquired funding acquisition. All authors have read and agreed to the published version of the manuscript.

## Supporting information



Supporting Information

## Data Availability

The data that support the findings of this study are available from the corresponding author upon reasonable request.
