# Supplementary material for: Syntaxin 17 Translocation Mediated Mitophagy Switching Drives Hyperglycemia‐Induced Vascular Injury
Source: Adv Sci (Weinh). 2025 Mar 26;12(19):2414960. doi: 10.1002/advs.202414960 (PMC12097103; doi:10.1002/advs.202414960)
Supplement: Supplementary file 1 — Supporting Information [file ADVS-12-2414960-s001.docx]

Supporting Information

**Syntaxin 17 Translocation Mediated Mitophagy Switching Drives Hyperglycemia-Induced Vascular Injury**

Anqi Luo, Rui Wang, Jingwen Gong, Shuting Wang, Chuan Yun, Zongcun Chen, Yanan Jiang, Xiaoquan Liu*, Haofu Dai*, Haochen Liu* and Yunsi Zheng*

Supporting Information includes:

Supplementary Figures S1-S5


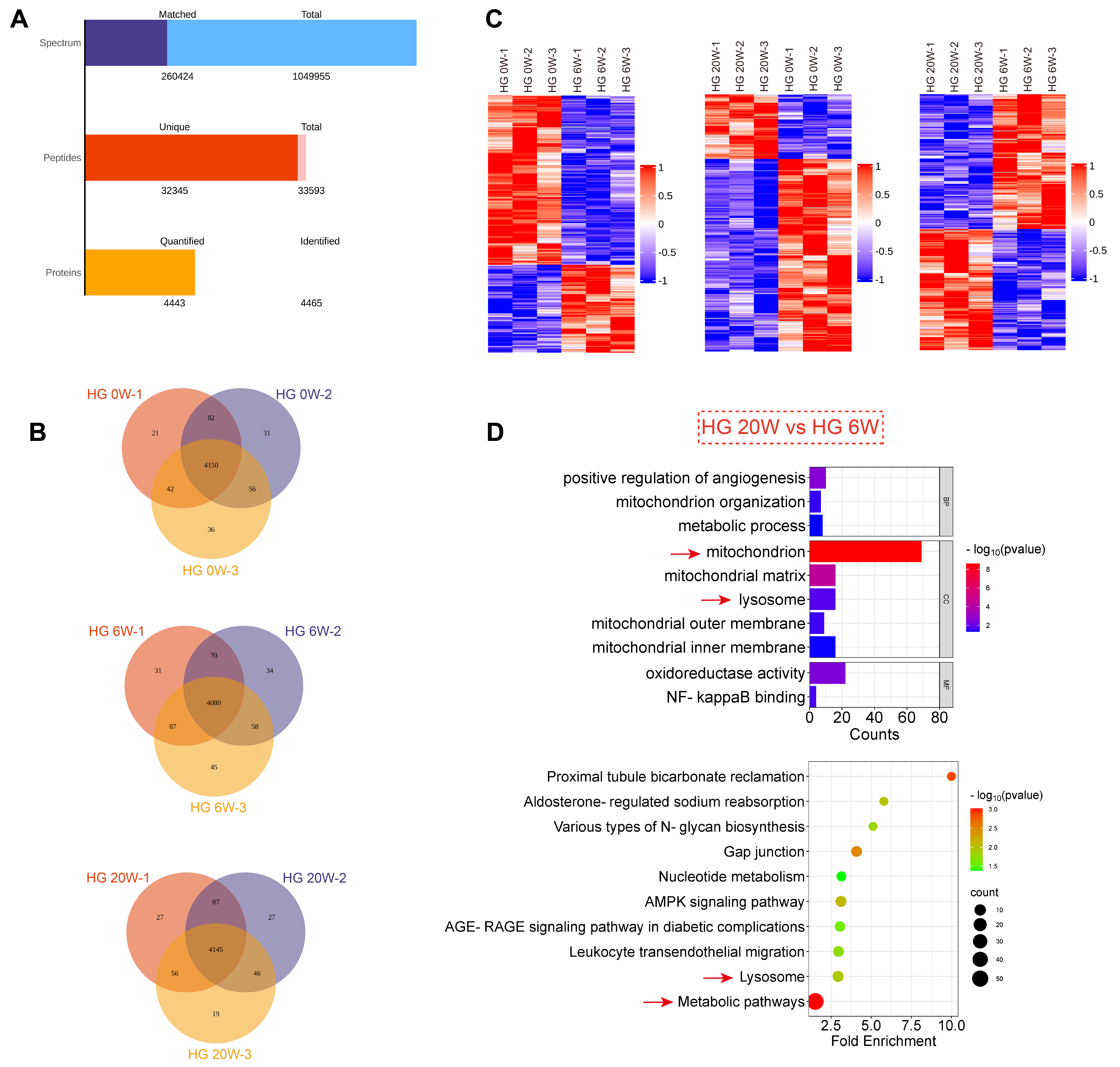


**Figure S1.** Quantitative proteome analysis. Male ApoE-/- mice (6 weeks old) were injected with STZ to induce hyperglycemia. Mouse aortas were collected at 0 weeks (HG 0W), 6 weeks (HG 6W), and 20 weeks (HG 20W) following hyperglycemia exposure. A) Bar chart of protein identification and quantification. n = 3. B) Quantitative Venn diagrams showing proteins shared among samples from the HG 0W group. Quantitative Venn diagrams showing proteins shared among samples from the HG 6W group. Quantitative Venn diagrams showing proteins shared among samples from the HG 20W group. n = 3. C) Cluster analysis chart depicting significantly differentially expressed proteins. n = 3. D) Bar and bubble charts showing the Gene Ontology (GO) analysis (categorized by biological process [BP], cellular component [CC], and molecular function [MF]) and the Kyoto Encyclopedia of Genes and Genomes (KEGG) pathway enrichment analysis of differentially expressed proteins (HG 20W vs. HG 6W). n = 3.


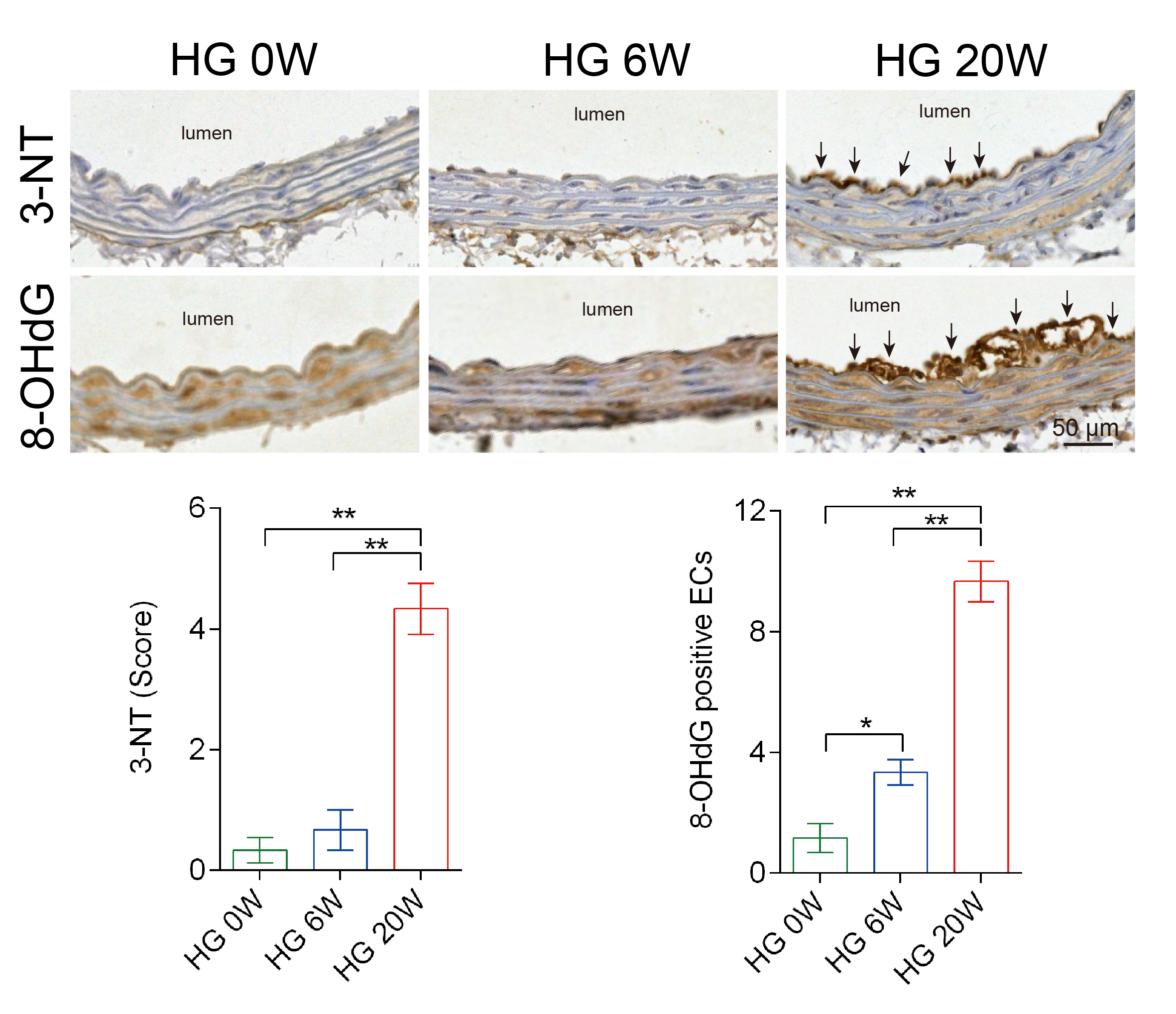


**Figure S2.** Immunohistochemical analysis of oxidative stress markers in aortic endothelium. Male ApoE-/- mice (6 weeks old) were injected with STZ to induce hyperglycemia. Mouse aortas were collected at 0 weeks (HG 0W), 6 weeks (HG 6W), and 20 weeks (HG 20W) following hyperglycemia exposure. Representative images and quantification of immunohistochemical staining for 8-OHdG and 3-NT in abdominal aortic sections. Scale bar = 50 μm. n = 6. Data are expressed as mean ± SEM. *p < 0.05; **p < 0.01.


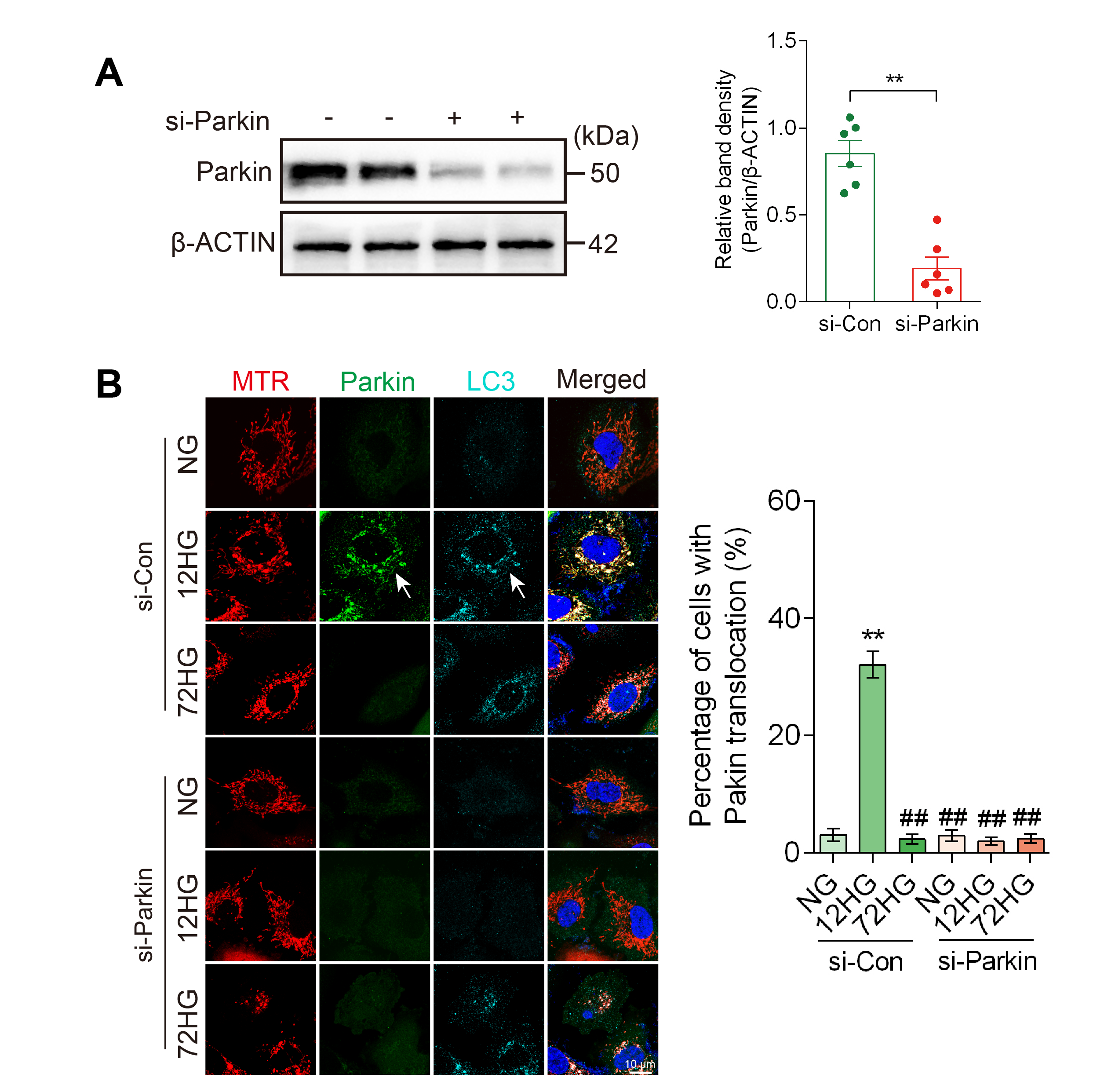


**Figure S3.** Parkin silencing reduced Parkin-mediated mitophagy. HUVECs were transfected with small interfering RNA (siRNA) against control (si-Con) or Parkin (si-Parkin) and then exposed to normal glucose (NG group), 12 hours of high glucose (12HG group), or 72 hours of high glucose (72HG group). A) Representative Western blot bands and relative band intensities of Parkin expression in the si-Con and si-Parkin groups. n = 6. B) HUVECs were stained with MitoTracker (red) and analyzed by immunofluorescence microscopy for Parkin (green) and LC3 (cyan). Nuclei were stained with Hoechst (blue). The bar chart shows the percentage of cells with Parkin translocation. Scale bar = 10 μm. n = 6. Data are expressed as mean ± SEM. *p < 0.05; **p < 0.01 vs. NG + si-Con group; #p < 0.05, ##p < 0.01 vs. 12HG + si-Con group.


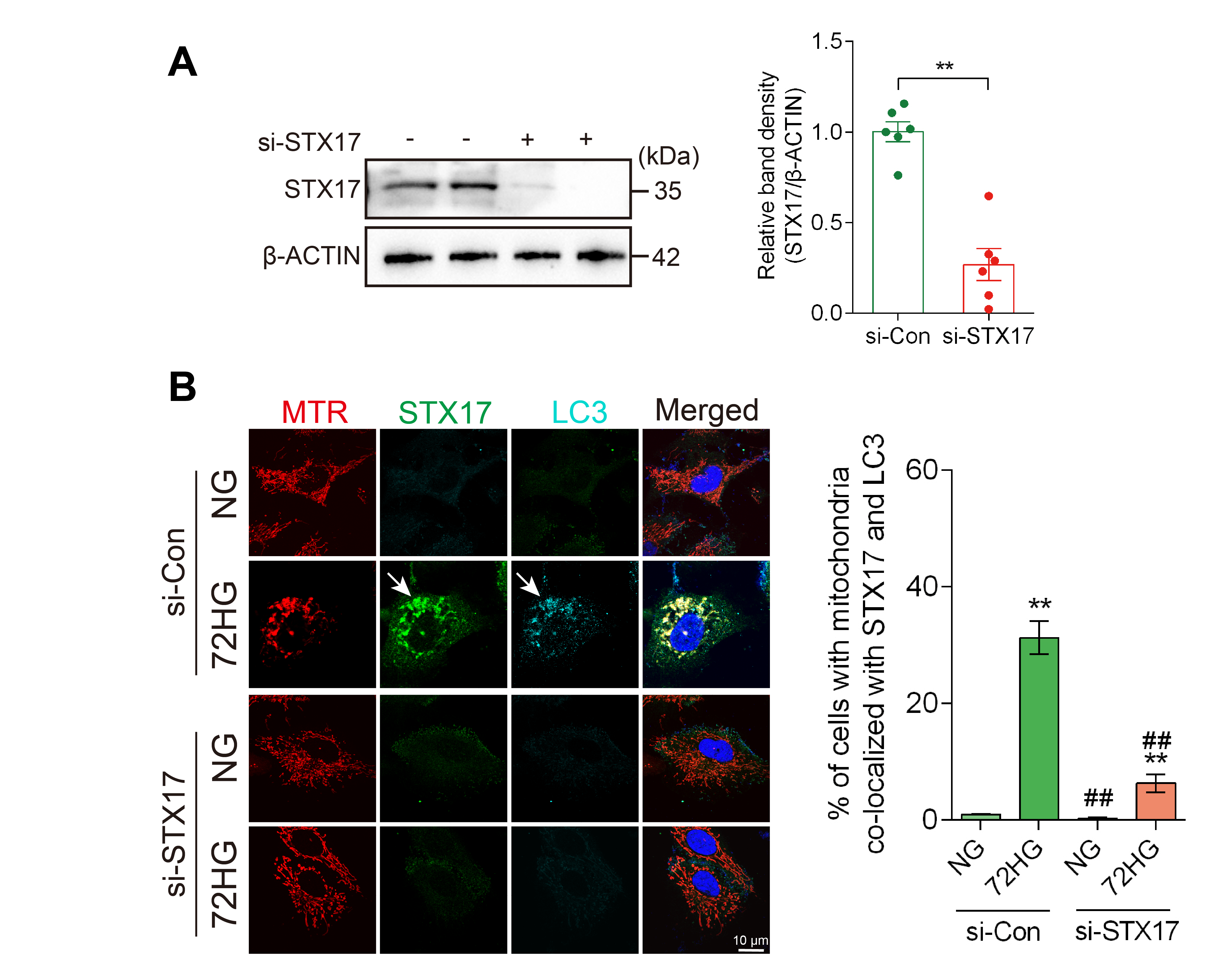


**Figure S4.** STX17 silencing reduced STX17-mediated mitophagy. HUVECs were transfected with small interfering RNA (siRNA) against control (si-Con) or STX17 (si-STX17) and then exposed to normal glucose (NG group) and 72 hours of high glucose (72HG group). A) Representative Western blot bands and relative band intensities of STX17 expression in the si-Con and si-STX17 groups. n = 6. B) HUVECs were stained with MitoTracker (red) and analyzed by immunofluorescence microscopy for STX17 (green) and LC3 (cyan), with nuclei stained with Hoechst (blue). The bar chart shows the percentage of cells with mitochondria co-localized with STX17 and LC3. Scale bar = 10 μm. n = 6. Data are expressed as mean ± SEM. *p < 0.05; **p < 0.01 vs. NG + si-Con group; #p < 0.05, ##p < 0.01 vs. 72HG + si-Con group.


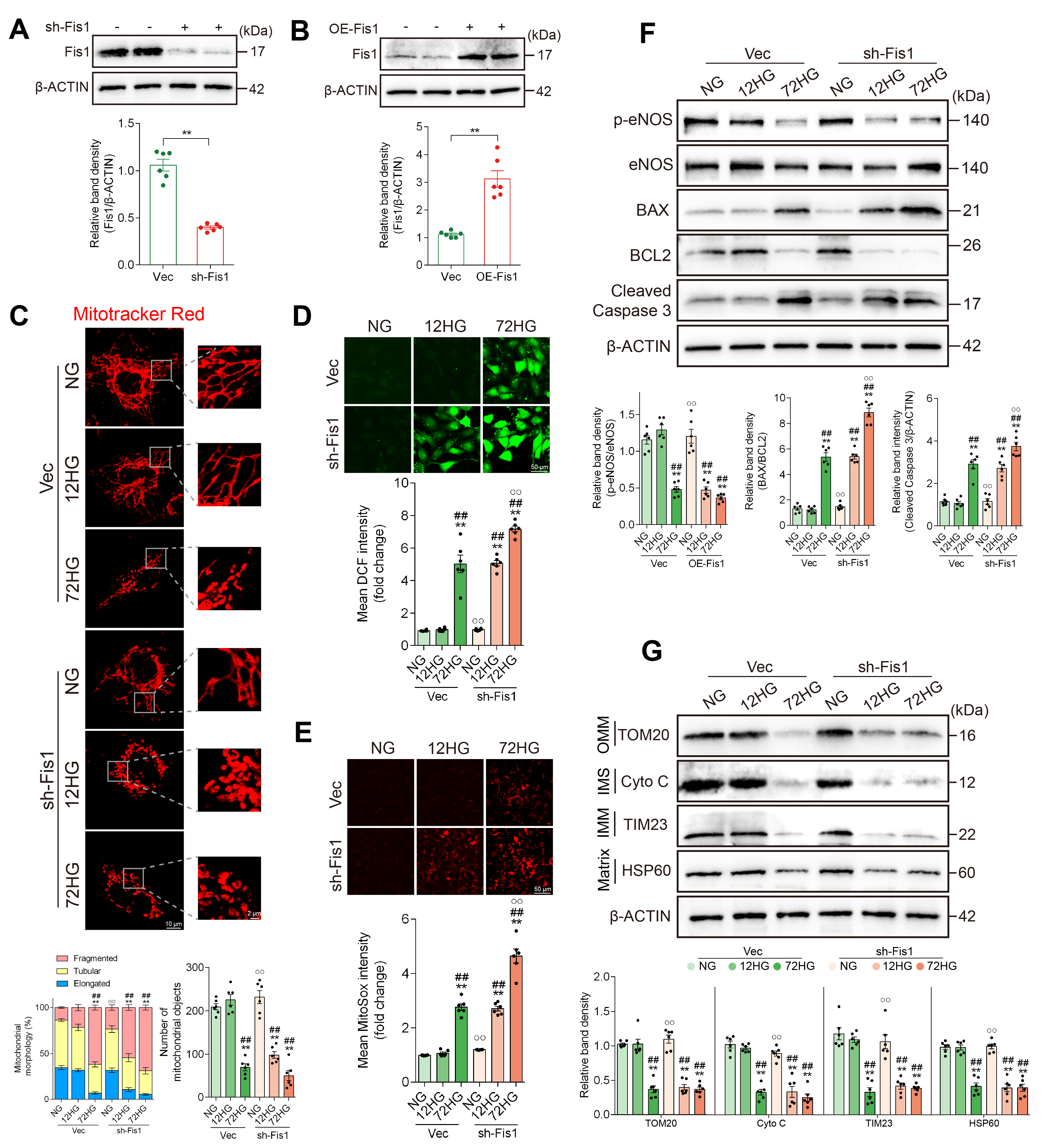


**Figure S5.** Fis1 silencing exacerbated high-glucose-induced vascular endothelial injury in HUVECs exposed to long-term high-glucose. A) HUVEC cell lines with Fis1 silencing (sh-Fis1) were constructed using lentivirus-mediated transduction. Representative Western blot bands and relative band intensities of Fis1 expression in the Vec and sh-Fis1 groups. n = 6. B) HUVEC cell lines with Fis1 overexpression (OE-Fis1) were constructed using lentivirus-mediated transduction. Representative Western blot bands and relative band intensities of Fis1 expression in the Vec and OE-Fis1 groups. n = 6. C-G) HUVEC cell lines with Fis1 silencing (sh-Fis1) were constructed using lentivirus-mediated transduction and then exposed to normal glucose (NG group), 12 hours of high glucose (12HG group), and 72 hours of high glucose (72HG group). C) HUVECs were stained with MitoTracker Red. Quantitative analysis of mitochondrial length and total number of fragmented, tubular, and elongated mitochondrial structures were quantitatively analyzed in each group. Scale bar = 10 μm. n = 6. D-E) ROS and mitochondrial ROS production were measured by DCF and MitoSOX staining, respectively. Scale bar = 50 μm. n = 6. F) Representative Western blot bands and relative band intensity of BAX, BCL2, cleaved caspase-3, p-eNOS and eNOS in HUVECs. n = 6. G) Representative Western blot bands and relative band intensities of TOM20, Cyto C, TIM23, and HSP60, representing proteins from the outer mitochondrial membrane (OMM), intermembrane space (IMS), inner mitochondrial membrane (IMM), and matrix. n = 6. Data are expressed as mean ± SEM. *p < 0.05; **p < 0.01 vs. NG + Vec group; #p < 0.05, ##p < 0.01 vs. 12HG + Vec group; ○p < 0.05, ○○p < 0.01 vs. 72HG + Vec group. Vec, vector.
